# Essential role of ATP6AP2 enrichment in caveolae/lipid raft microdomains for the induction of neuronal differentiation of stem cells

**DOI:** 10.1186/s13287-018-0862-9

**Published:** 2018-05-11

**Authors:** Nehman Makdissy, Katia Haddad, Jeanne D’arc AlBacha, Diana Chaker, Bassel Ismail, Albert Azar, Ghada Oreibi, David Ayoub, Ibrahim Achkar, Didier Quilliot, Ziad Fajloun

**Affiliations:** 1 Department of Biology, Lebanese University, Faculty of Sciences III, Kobbe, Lebanon; 2 Doctoral School for Sciences and Technology, Azm Center for the Research in Biotechnology and its Applications, Lebanese University, Tripoli, Lebanon; 3 Doctoral School for Sciences and Technology, Faculty of Sciences I, Lebanese University, Hadath, Lebanon; 4 Reviva Regenerative Medicine Center, Human Genetic Center, Middle East Institute of Health Hospital, Bsalim, Lebanon; 5 Ayoub Clinic Lebanon and Department of Neuroloradiology, Limoges University Hospital, EA3842 Limoges, Lebanon; 6 Achkar Clinics, St. Elie Center, Antelias, Lebanon; 7 Diabetologia-Endocrinology & Nutrition, CHRU Nancy, INSERM 954, University Henri Poincaré, Faculty of Medicine, Nancy, France

**Keywords:** ATP6AP2, Caveolae, Caveolin, Exosomes, Flotillin, Lipid rafts, Wnt signaling, Neural differentiation, Renin, Stem cells

## Abstract

**Background:**

The subcellular distribution of prorenin receptor and adaptor protein ATP6AP2 may affect neurogenesis. In this study, we hypothesized that ATP6AP2 expression and subcellular relocalization from caveolae/lipid raft microdomains (CLR-Ms) to intracellular sites may correlate with neuronal differentiation (Neu-*Dif*) of adipose-derived mesenchymal stem cells (ADSCs).

**Methods:**

Human ADSCs isolated from 24 healthy donors and 24 patients with neurological disorders (ND) were cultured and induced for Neu-*Dif*. The mechanism of action of ATP6AP2 and the impact of its localization within the plasma membrane (particularly CLR-Ms) and intracellular sites on several pathways (mitogen-activated protein kinase, Wnt(s) signaling and others) and intracellular calcium and exosome release were evaluated. The impact of CLR-Ms on ATP6AP2 or vice versa was determined by pharmacological disruption of CLR-Ms or siATP6AP2 assays.

**Results:**

In patients with ND, loss of ATP6AP2 from CLR-Ms correlated with an inhibition of Neu-*Dif* and signaling. However, its relocalization in CLR-Ms was positively correlated to induction of Neu-*Dif* in healthy subjects*.* An apparent switch from canonical to noncanonical Wnt signaling as well as from caveolin to flotillin occurs concurrently with the increases of ATP6AP2 expression during neurogenesis. Stimulation by renin activates ERK/JNK/CREB/c-Jun but failed to induce β-catenin. Wnt5a enhanced the renin-induced JNK responsiveness. Gα proteins crosslink ATP6AP2 to caveolin where a switch from Gαi to Gαq is necessary for Neu-*Dif*. In ATP6AP2-enriched CLR-Ms, the release of exosomes was induced dependently from the intracellular Ca^2+^ and Gαq. Pharmacological disruption of CLR-M formation/stability impairs both ATP6AP2 localization and Neu-*Dif* in addition to reducing exosome release, indicating an essential role of ATP6AP2 enrichment in CLR-Ms for the induction of Neu-*Dif*. The mechanism is dependent on CLR-M dynamics, particularly the membrane fluidity. Knockdown of ATP6AP2 inhibited Neu-*Dif* but increased astrocytic-*Dif*, depleted ATP6AP2/flotillin/Gαq but accumulated caveolin/Gαi in CLR-Ms, and blocked the activation of JNK/ERK/c-Jun/CREB/exosome release. siATP6AP2 cells treated with sphingomyelinase/methyl-β-cyclodextrin reversed the levels of caveolin/flotillin in CLR-Ms but did not induce Neu-*Dif*, indicating the crucial relocalization of ATP6AP2 in CLR-Ms for neurogenesis. Treatment of ND-derived cells with nSMase showed reversibility in ATP6AP2 abundance in CLR-Ms and enhanced Neu-*Dif.*

**Conclusions:**

This study gives evidence of the determinant role of CLR-M ATP6AP2 localization for neuronal and oligodendrocyte differentiation involving mechanisms of switches from Gαi/caveolin/canonical to Gαq/flotillin/PCP, the ERK/JNK pathway and Ca^2+^-dependent release of exosomes and as a potential target of drug therapy for neurodegenerative disorders.

**Electronic supplementary material:**

The online version of this article (10.1186/s13287-018-0862-9) contains supplementary material, which is available to authorized users.

## Background

MSCs are multipotent stromal cells with the potential to differentiate into neural cells, involving several signaling pathways. An important role for expression of the renin–angiotensin system (RAS) has been reported in the regulation of human MSC differentiation [1]. ATP6AP2 was described as a renin and prorenin receptor ((P)RR) exerting a RAS-related function but also as an adaptor protein between V-ATPase and Wnt receptor complex [2]. Binding of prorenin or renin to the extracellular domain of ATP6AP2 activates the RAS, leading to the production of angiotensin II and other pathways, such as the MAPK pathway, in particular ERK [3]. The beginning of the discovery of ATP6AP2 as an essential component for RAS was followed by that of its expression in the brain [4, 5]. In XPDS patients, a decrease of ATP6AP2 was observed in the brain [6]. In the mouse and fly, conditional depletion of ATP6AP2 induces cognitive impairment and neurodegeneration [7], and regulation of adult hippocampal neurogenesis via Wnt/PCP/β-catenin pathways. In *Xenopus*, ATP6AP2 is essential for mediating Wnt signaling during early central nervous system development, including neural patterning [8]. Thereby, emerging studies have drawn attention to the importance of ATP6AP2 in cognitive processes and brain development while suggesting a vital role of the RAS in learning and memory functions. However, little is known about the importance of the colocalization of ATP6AP2 with key signaling factors, and even rarer are studies about its relocalization and its relationship with plasma membrane microdomains particularly in MSCs.

Previous studies underline the combination role of ATP6AP2 and other factors, among them, for example, vascular endothelial growth factor (VEGF), a growth factor secreted by MSCs [9], known to possess neurogenic effects, promote NSC activation and regulate their conversion into progenitor cells and neurons [10]. Importantly, colocalization of ATP6AP2 and VEGF has been reported in human retinal microvascular endothelial cells [11]. Moreover, ATP6AP2 interacting with microRNA-152 (a potential regulator of ATP6AP2) regulates downstream VEGF expression [12]. Recent evidence has also shown that neuronal differentiation (Neu-*Dif*) of neural progenitor cells can be regulated by caveolin (CAV) proteins, components of the microdomain lipid rafts (MLRs), via modulation of a variety of neuronal intracellular signaling pathways, among them VEGF, ERK, Akt and Stat3 [13, 14]. All three CAV isoforms are expressed in neurons and immature neuronal-like cells lose CAV expression upon differentiation [15–18]. In fact, MLRs enriched in sphingolipids, cholesterol, scaffolding proteins and a multitude of receptors including neurotransmitter receptors serve as a platform for neuronal signal transduction and cytoskeletal organization by interaction with CAV [19]. Among the proteins in MLRs, the integral membrane flotillin (FLOT) may represent a functional analogue of CAV in CAV-negative or downregulated cells; in fact, FLOT is highly expressed in cells that lack CAV (e.g., neurons) [20]. FLOT-1/FLOT-2 define raft-related microdomain signaling centers in neurons and astrocytes [21]. Any perturbation in MLRs can affect the cell autophagy or the release of exosomes affecting FLOTs and CAVs, and may have significant impacts on Wnt signaling as well as secreted inflammatory cytokines [22, 23]. In addition, several lines of evidence suggest that V-ATPase is a major component of the MLR fraction of synaptic plasma membrane and synaptic vesicle membrane [24].

Taking into consideration the potential role of ATP6AP2 in neuronal development, several points remain to be clarified concerning its role in MSCs during neurogenesis: ATP6AP2 has not yet been identified in MSCs, and the subcellular distribution of (P)RR in MSCs and the relationship with MLRs remain to be elucidated. Therefore, we were interested in this study to isolate human adipose-derived mesenchymal stem cells (hADSCs) and to evaluate the relationship between ATP6AP2 and these microdomains, studying the mechanisms involving Wnt/Gα proteins/intracellular calcium/exosome release signaling pathways. The role of ATP6AP2 was validated with the use of gene knockdown ADSCs. A neurological diseases group of patients was compared to a healthy group of subjects.

## Methods

### Subjects

**Fig. 1:**
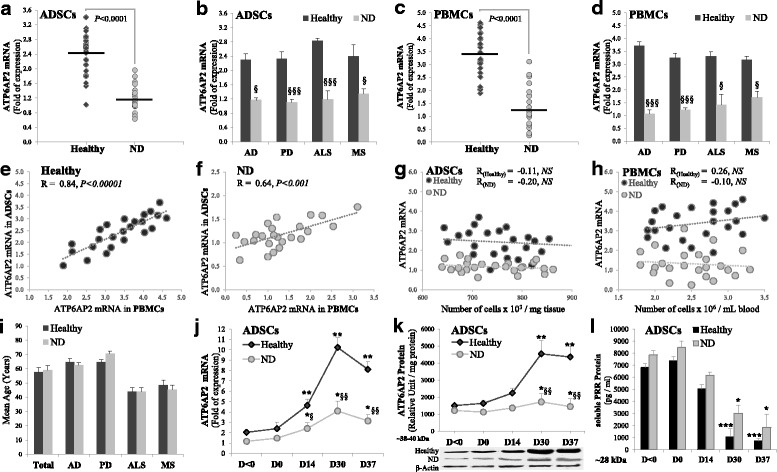
Expression of ATP6AP2 in PBMCs and ADSCs. PBMCs and ADSCs isolated from healthy or ND subjects. mRNA expression determined by RT-qPCR and normalized to GAPDH. Quantitative levels of ATP6AP2 mRNA in undifferentiated ADSCs (**a**, **b**) or PBMCs (**c**, **d**). Correlations of expression of ATP6AP2 between ADSCs and PBMCs in healthy (**e**) or ND (**f**) subjects. Correlations with isolated number of cells in ADSCs (**g**) or PBMCs (**h**). Distribution of population as a function of donor’s age (**i**). ADSCs differentiated into NLCs for 37 days as indicated in Methods and levels of mRNA (**j**), total (**k**) and soluble (**l**) proteins shown. Undifferentiated cells analyzed at day < 0 (**a**–**h**). Differentiated cells analyzed at D < 0/D0/D14/D30/D37 (**j–l**). *^§^*P* < 0.05,**^§§^*P* < 0.001,***^§§§^*P* < 0.0005: *D0/14/30/37 vs D < 0, ^§^ND vs healthy. AD Alzheimer disease, ADSC adipose-derived mesenchymal stem cell, ALS amyotrophic lateral sclerosis, D day, MS multiple sclerosis, ND neurodegenerative disease, NS not significant, PBMC peripheral blood mononuclear cell, PD, Parkinson disease

Healthy subjects (*n* = 24, 57.8 ± 3.1 years old) were enrolled in the study if they had no notable pathologic history in particular neurological diseases. Subjects with confirmed diagnosis of ND (*n* = 24, 58.9 ± 3.4 years old) (Alzheimer’s disease (AD), *n* = 6; Parkinson’s disease (PD), *n* = 9; amyotrophic lateral sclerosis (ALS), *n* = 4; multiple sclerosis (MS), *n* = 5) were considered for this study as ND patients. The selection of subjects’ age was group matched (healthy vs ND) (Fig. 1). The control group consisted of healthy volunteers matched to the ND groups for age and sex. Subjects were excluded from the two groups if they had histories of neurologic conditions including moderate or severe head injury, stroke, cerebral or bone damage or malignancies, brain abnormalities, learning disability, major medical or psychiatric illness in the previous 6 months, any metabolic/cardiovascular disease or evidence of cardiac/renal damage or malignancies, alcohol, loss of weight during the last 2 years, chemotherapy or immunosuppressive therapy.

### Isolation and culture of peripheral blood mononuclear cells

Fresh peripheral blood (20 ml) was collected from healthy and ND subjects. Peripheral blood mononuclear cells (PBMCs) were separated and cultured according to AlBacha et al. [25]. No significant variations on the number of isolated cells were obtained between healthy and ND subjects (data not shown).

### Isolation and culture of hADSCs

Samples of human adipose tissue (200 ml or ~ 100–200 mg) were obtained by lipoaspiration or biopsy from abdominal subcutaneous fat, and then processed for the isolation of SVF and culture of ADSCs as described previously [26]. The differentiation was induced at the 3rd passage after confirming the absence of any chromosomal abnormality as determined by karyotyping. No significant variations in the number of isolated cells were obtained between healthy and ND subjects (Fig. 1).

### Neuronal induction of hMSCs

This procedure consisted of two steps: induction of hMSCs to differentiate into neurosphere-like structures (NSPs), and final differentiation into neuron-like cells (NLCs). MSCs (10^6^ cells) were cultured in a neurobasal (NB) medium (Hyclone advanced basal medium stem) supplemented with 10% serum (Hyclone advanced stem cell growth supplement), 2% B27, 1% PSA at 37 °C and 5% CO_2_ (days 0–14). NB medium was supplemented with 20 ng/ml b-FGF and 20 ng/ml EGF for 16 days (days 14–30). Formed NSPs were collected at day 30 and then cultured to initiate final differentiation. NSPs (10^5^ cells) were cultured in (NB) medium supplemented with 10% serum, 2% B27, 1% PSA, 20 ng/ml β-NGF at 37 °C and 5% CO_2_ (days 30–37). Media were renewed every 3–4 days.

### Dosage of soluble (pro)renin receptor by ELISA

Soluble (pro)renin receptor (s(P)RR) was quantified using an ELISA kit (Immuno-Biological Laboratories) according to the instructions of the manufacturer. s(P)RR was detected by an HRP-conjugated antibody and TMB as chromogen.

### Membrane fluidity

The plasma membrane fluidity was assessed by steady-state fluorescence polarization using the lipophilic fluorescent probe DPH. The measured fluorescence anisotropy, inversely related to fluidity, was determined as previously described by Makdissy et al. [27].

### Cell fractionation and isolation of caveolae/lipid rafts

Caveolae/lipid raft MLRs were prepared according to the method of Makdissy et al. [27] adapted for MSCs, NSPs and NLCs. In total, 12–13 fractions were obtained, subdivided into: nuclear fraction, postnuclear fraction (which was depleted from nuclear and plasma membranes), microsomal fraction and plasma membranes (total or fractioned into the caveolae/lipid raft plasma membrane microdomain (CLR-M) fraction and the noncaveolae/nonlipid raft plasma membrane microdomain (N-CLR-M) fraction). To ensure the absence of nuclear and microsomal membrane contamination in the plasma membrane fraction, the obtained fractions were analyzed for the presence of nuclear protein nucleoporin p62 and largely Golgi-localized protein TGN38 (H-122 (sc-25523) and C-15 (sc-27680)) (Santa Cruz) (Additional file 1: Table SI1).

### Cell fractionation and isolation of intracellular organelles

After isolation of cytoplasmic and nuclear fractions from cultured cells, the intracellular organelle fraction was isolated by OptiPrep density gradient and sucrose. The isolation of endoplasmic reticulum (ER) Golgi and lysosome fractions (EL0100, GL0010, and LYSISO1, respectively; Sigma) was performed according to the manufacturer’s instructions. Organelle markers were used to identify the intracellular components: ER (Calnexin, TRAPα), Golgi (TGN38, GM130), endosome (EEA1), lysosome (LAMP1) and nuclear (Nucleoporin, Lamin A).

### Exosome purification

Exosomes were isolated from the culture media of 10^7^ cells according to the method described previously by Théry et al. [28]. The purity of the exosomes was evaluated by flow cytometry determining the percentages of expression of specific tetraspanin exosome biomarkers (CD9, CD63 and CD81) and FLOT, and the absence of the ER marker (calnexin). Released exosomes were quantitated by measuring the activity of their specific enzyme: acetylcholinesterase (AChE). The exosome fraction was suspended in PBS (1:4, v:v) and incubated with DTNB (0.1 mM) and acetylthiocholine (1.25 mM) in a final volume of 1 ml at 37 °C, and the change in absorbance at 412 nm was determined continuously at 10/20/30/60/180 min. The data represent the enzymatic activity at 30 min of incubation (saturation reached its maximum at 20 min).

### Magnetic separation of TUJ1^(+)^/O4^(+)^/GFAP^(+)^ cells

hADSCs derived from healthy and ND subjects were cultured and differentiated as described previously. Purifications of TUJ1^(+)^/O4^(+)^/GFAP^(+)^ cells were performed on the mixed population at day < 0 (before the induction of the differentiation) and at day 37 (end of the Neu-*Dif*). Then, 20 × 10^7^ total cells were collected and subjected to purification using MACS technology (Miltenyi) according to the manufacturer’s instructions. Cells were stained for 30 min in the dark with the corresponding fluorochrome-conjugated primary antibody (monoclonal anti-TUBB3-FITC antibody (for TUJ1 staining; Sigma-Aldrich), monoclonal anti-O4-APC antibody (for O4 staining; Miltenyi) and monoclonal anti-GFAP-PE antibody (for GFAP staining; Miltenyi)); a first measure on a sample of the mixed population was done by flow cytometry for determination of the percentage of cells which may express these markers. Therefore, we proceeded with targeted-cell purification for 15 min at 4 °C: to obtain a single-cell suspension before magnetic labeling, cells were passed through 70-μm nylon mesh (Miltenyi) to remove cell clumps which may clog the column. Subsequently, the obtained single-cell suspension was magnetically labeled firstly with anti-FITC Microbeads (Miltenyi) for the purification of TUJ1. The cell suspension was loaded on a MACS column which was placed in the magnetic field of a MACS separator. The magnetically labeled TUJ1^(+)^ cells were retained in the column while the unlabeled cells ran through (negative fraction designated N1: this cell fraction was thus depleted from TUJ1^(+)^). After removing the column from the magnetic field, the magnetically retained TUJ^(+)^ cells were eluted as the positively selected TUJ^(+)^ cell fraction (positive fraction designated P1). Next, the negative fraction N1 was then labeled with anti-APC Microbeads (Miltenyi) for the purification of O4; the magnetically labeled O4^(+)^ cells were purified as described previously to obtain a P2 fraction, while the unlabeled cells constituted the negative fraction N2 which was depleted of TUJ^(+)^ and O4^(+)^. Finally, N2 was labeled with anti-PE Microbeads (Miltenyi) for purification of GFAP; the same procedure and elution as described previously was used to obtain P3 enriched with GFAP^(+)^ cells. To increase the purity, the positively selected cell fractions were separated over a second column. After fluorescent labeling, samples of the obtained positive and negative fractions were reanalyzed by flow cytometry to assess purity and analyze the immunophenotyping of the cells.

### Flow cytometry analysis

Cells were stained with human anti-CD34(Vioblue), CD45(FITC), CD73(PE), CD90(APC) and CD105(Vioblue) antibodies (Miltenyi-Biotech), TUJ1 (Covance or Sigma-Aldrich), NeuN and GFAP (Abcam or Miltenyi) and O4 (Sigma). Purified exosomes were fixed and incubated with human anti-CD9(PE), anti-CD63(APC) and anti-CD81(PerCP) antibodies (Miltenyi), FLOT-1/FLOT-2 and β-catenin (Santa-Cruz) and calnexin (Abcam). Appropriate APC/FITC/PE-conjugated secondary antibodies were used in cases of staining with primary unconjugated antibodies. The flow cytometry protocol and analysis was performed following supplier instructions on an MACSQuant analyzer (Miltenyi). Isotype controls and automated compensation were settled to minimize false positive fluorescence and spectral overlap of fluorochromes respectively. Cell viability and apoptosis were assessed by the 7AAD/AnnexinV/PI assay.

### Immunostaining and fluorescence microscopy analysis

At day 37 of Neu-*Dif*, cells were seeded on round glass coverslips coated with poly-l-lysine (0.01%), fixed in 4% paraformaldehyde (20 min) and then washed twice with PBS (5 min). Three sequential immunostainings were performed with three different antibodies: TUJ1, GFAP and O4. Immunostaining was performed after blocking in 3% normal goat serum and adding primary mouse monoclonal TUJ1 (1/1000), rabbit polyclonal IgG anti-GFAP (1/800) and mouse monoclonal anti-O4 (1/1000). The secondary antibodies were ATTO α-mouse IgG Alexa 488 (1/500), AHO 647 α-rabbit (1/500) and mouse IgM 488 (1/1000). Slides were treated with VECTASHIELD mounting Medium with DAPI and viewed on a SP5 inverted confocal microscope. Images were captured and analyzed on Leica software.

### RT-qPCR

RNA was isolated with the RNAspin Mini kit (GE Healthcare) according to the manufacturer’s instructions and DNase digestion was performed with the RNase-Free DNase Set (Qiagen). The yield and purity of RNA were assessed spectrophotometrically and the integrity by agarose gel electrophoresis. First-strand cDNA was synthesized from 400 ng RNA using Superscript III reverse transcriptase (Invitrogen) according to the manufacturer’s protocol. cDNA (25 ng) relative to total RNA was amplified in 20 μl using VeriQuest Fast SYBR Green qPCR Master Mix (Affymetrix). Thermal cycling was performed on a LightCycler 2.0 (Roche): 1 cycle at 50 °C/2 min, 1 cycle at 95 °C/5 min, 45 cycles at 95°C/3 s and 60 °C/30 s. The primers are presented in Additional file 2: Table SI2. Relative changes in expression were calculated after normalization to GAPDH.

### ATP6AP2 siRNA transfection

MSCs at 70% of confluence were cultured (10^5^ cells) and transfected with Lipofectamine RNAiMAX (Thermofisher Scientific) according to the manufacturer’s instructions. MSCs were transfected with siRNA–Lipofectamine RNAiMAX complexes (respectively, 6000 pmol and 40 μl in 2 ml media) at day < 0 and the cells were induced to differentiate with the neuronal induction medium. To maintain the silencing effect, an additional dose of siRNAs was administered 7/14/28 days after the initial transfection. Silencing was validated by RT-qPCR. Cytotoxic effects were observed above 11,000 pmol of siRNA and if added with medium supplemented with antibiotics and high concentrations of serum (> 4%). Internal control of lipofectamine alone was used.

### Western blot analysis

The proteins were revealed with antibodies directed against ATP6AP2(HPA003156), ERK(sc-94), p-ERK(sc-7383), JNK(FL,sc-571), p-JNK(G-7,sc-6254), CREB-1(sc-58), p-CREB-1(sc-7978), c-Jun(sc-74,543), p-c-Jun(sc-822), β-catenin(c-7204, sc-7963), caveolin(sc-894), flotillin(sc-30,750), Gαs/olf(sc-55,545), Gαi_1–3_(sc-13,534), Gαq(sc-136,181), nucleoporin p62(sc-25,523) and TGN38(sc-27,680). SDS-PAGE analysis was performed as described previously by Makdissy et al. [27].

### Coimmunoprecipitation

Coimmunoprecipitation of ATP6AP2 and targeted proteins of the CLR-M fraction was performed according to Lu et al. [29]. Anti-ATP6AP2 or anti-caveolin or anti-Gαi/Gαq/Gαs antibodies were crosslinked with the magnetic beads and then incubated for 30 min with the CLR-M proteins. After collection, wash and elution of the beads, immunoblotting analysis was realized on the immunoprecipitated (IP) samples to identify the binding partner.

### Intracellular calcium assay

Free intracellular calcium concentration ([Ca^2+^]_i_) was determined using fura-2/AM as a calcium chelator. After serum-starving for 2 h, 10^5^ cells/ml were collected in BSA-KRH buffer and labeled with 160 nM fura2/AM for 45 min at 37 °C under 5% CO_2_. Then, cells were pelleted at 300 × *g* for 5 min, suspended in KRH buffer. After 15 min, CaCl_2_ (1.5 mM) was added to the sample and kept with the cells for 15 min. The total time elapsed between the end of the labeling and the first fluorescence determination was 35 min to allow full hydrolysis of the intracellular fura-2/AM ester. Fluorescence (*F*) was determined in a Perkin-Elmer LS-5 spectrofluorimeter (excitation 340–450 nm, emission 510 nm). All determinations were performed at 37 °C. [Ca^2+^]_i_ was calculated using the following equation:

$$ \left[{\mathrm{Ca}}^{2+}\right]={K}_{\mathrm{d}}\times \left[\left(R-{R}_{\mathrm{min}}\right)/\left({R}_{\mathrm{max}}-R\right)\right]\times \beta, $$

where *R* is the ratio of fluorescence of the sample at 340 and 380 nm (*F*_340_/*F*_380_); *R*_max_ and *R*_min_ represent the ratios for fura-2/AM at the same wavelengths of saturating Ca^2+^ determined by adding 1 μM ionomycin and minimal Ca^2+^ determined by adding 15 mM EGTA, respectively; *β* is the ratio of fluorescence of fura-2/AM at 380 nm in minimal and saturating Ca^2+^ (*F*_380 min_/*F*_380 max_); and *K*_d_ is the dissociation constant of fura-2/AM for Ca^2+^, assumed to be 224 nM at 37 °C. All measures were determined by deducing the autofluorescence for each sample. Results were expressed as the concentration of intracellular calcium (nM).

### Statistical analysis

Results are presented as the mean ± SEM of four independent experiments performed in duplicate and analyzed for statistical significance (on absolute values) using Student’s *t* test. For all statistical tests, *P* values were two-tailed and the level of significance was set at 0.05.

## Results

### Expression of ATP6AP2 in hPBMCs and hADSCs

We were interested to evaluate the expression of ATP6AP2 for the first time in primary undifferentiated hADSCs in comparison to circulated hPBMCs isolated from the peripheral blood of the same donor. We assessed whether ATP6AP2 mRNA expression may vary between healthy and ND subjects. Subjects considered for neurological disorders were designated the neurodegenerative diseases (ND) group, divided into four subgroups as described in Methods. All of the ND groups and subgroups were sex and age matched with the healthy ones. First, the expression of ATP6AP2 mRNA was evaluated in undifferentiated cells: ATP6AP2 mRNA was significantly reduced in hADSCs and hPBMCs derided from the ND group (Fig. 1a–d) in comparison to the healthy group. Positive correlations were found between ADSC-expressing ATP6AP2 and PBMC-expressing ATP6AP2 in healthy (*R* = 0.84, *P <* 0*.*00001) and ND (*R* = 0.64, *P <* 0*.*001) groups (Fig. 1e, f), indicating that the assessment of ATP6AP2 in hPBMCs can be considered a marker when the collection of adipose tissue is difficult to apply to donors with advanced ages. The moderate levels of ATP6AP2 mRNA expression in ND were not correlated to low levels of isolated cells, whether ADSCs or PBMCs (Fig. 1g, h), nor with age (data not shown).

Thus, we investigated the potential of hADSCs to differentiate into neural lineages during 37 days as indicated in Methods and assessed the expression of ATP6AP2 during this Neu-*Dif*: ATP6Ap2 mRNA and protein reached maximum levels of expression at day 30 of the differentiation; significantly lower levels of expressions were observed in the ND group (Fig. 1j, k). The decrease in ATP6AP2 protein in ND led us to investigate whether this decrement is due to an increase in the activity of the furin, the enzyme that cleaves *PRR* intracellularly in the trans-Golgi to generate soluble prorenin receptor (s(P)RR), which would subsequently be secreted by the cell. The quantification of s(P)RR in the culture media showed no significant variations between healthy and ND subjects, indicating that the observed decreases in cellular proteins could be predominantly related to the transcriptional activity and not to an elevated furin activity, and indicate that undifferentiated cells had elevated furin activity (Fig. 1l).

Analysis between subgroups within the ND population did not alter the represented results: in fact, all of the obtained data were similar and no significant variations were observed between the dual subgroups (AD + PD) or (ALS + MS) and the controls.

### Neurogenic potential of hADSCs

**Fig. 2:**
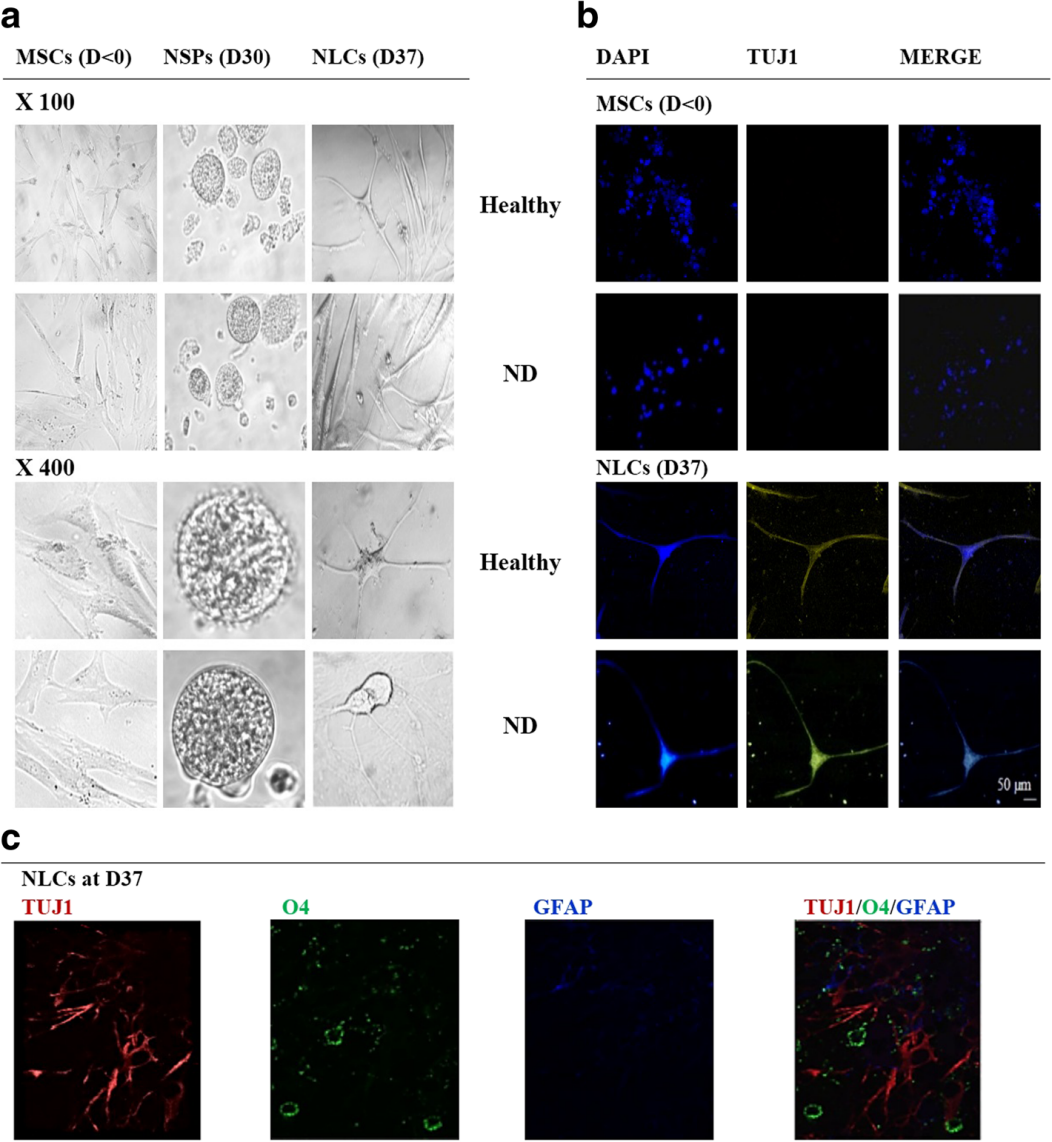
Morphological cell changes during Neu-*Dif*. hADSCs isolated from healthy or ND subjects and differentiated for 37 days as indicated in Methods. Cell morphology at days 0 (MSCs), 21 (NSPs) and 37 (NLCs) of neural differentiation showing progeny of single cell-derived neurosphere and neural-like structures (**a**). Immunostaining assays of MSCs (at day < 0) and NLCs (at day 37) stained with TUJ1 (yellow) and nuclei counterstained with DAPI (blue) (**b**). Triple labeling of the single-cell suspension fully differentiated at D37 stained with TUJ1 (red), O4 (green) and GFAP (blue) (**c**). D day, DAPI 4′,6-diamidino-2-phenylindole, MSC mesenchymal stem cell, ND neurodegenerative disease, NLC neuron-like cell, NSP neurosphere-like structure

Cell morphology changes from a fusiform fibroblast type (MSC) to a neurocyte-like cell shaped in neurospheres (NSP) and getting a neural-like cell (NLC) at the end of ADSC differentiation (Fig. 2a). Similar morphologies were observed between cells derived from healthy and ND subjects.

The immunophenotyping of the cells was assessed at days < 0, 30 and 37 of the Neu-*Dif* by flow cytometry; we realized multiplex labeling of the single-cell suspension of the same sample using different fluorochrome-conjugated antibodies. A switch from the mesenchymal phenotype (CD34^−^/CD45^−^/CD73^+^/CD90^+^/CD105^+^) in undifferentiated MSCs to almost its absence (CD34^−^/CD45^−^/CD73^−^/CD90^−^/CD105^−^) indicated a significant cell differentiation; the apoptotic index indicated that there were no significant intragroup and intergroup variations between MSCs, NSPs and NLCs (Table 1).

**Table 1:** Immunophenotyping, apoptotic index and distribution of neurogenic markers on unpurified mixed population

	MSCs Day < 0	NSPs Day 30	NLCs Day 37
Differentiation of hADSCs in healthy subjects			
CD34^(+)^	1.26 ± 2.01	0.18 ± 1.64	0.51 ± 2.60
CD45^(+)^	1.28 ± 1.17	0.71 ± 0.92	0.07 ± 1.34
CD73^(+)^	90.12 ± 5.52	24.51 ± 10.34 ^*^	6.77 ± 3.68 ^**^
CD90^(+)^	95.59 ± 6.80	4.07 ± 3.76 ^**^	1.94 ± 2.1 ^***^
CD105^(+)^	84.33 ± 5.33	18.68 ± 4.51 ^*^	1.26 ± 1.90 ^***^
Annexin V^(−)^/PI^(−)^/7AAD^(−)^ (viable cells)	98.97 ± 0.71	96.20 ± 1.25	96.85 ± 0.83
Annexin V^(+)^/PI^(−)^/7AAD^(−)^ (early-apoptotic cells)	0.76 ± 0.90	2.11 ± 1.01	2.03 ± 1.04
Annexin V^(+)^/PI^(+)^/7AAD^(+)^ (late apoptotic, necrotic cells)	0.27 ± 0.71	1.69 ± 0.99	1.12 ± 1.10
NeuN	1.62 ± 1.54	58.14 ± 3.32 ^**^	78.12 ± 6.28 ^**^
TUJ1	4.49 ± 1.62	60.48 ± 5.25 ^**^	89.09 ± 8.67 ^**^
O4	1.28 ± 2.40	28.16 ± 4.11^*^	26.33 ± 3.29 ^*^
GFAP	24.14 ± 2.58	4.26 ± 1.84 ^**^	1.20 ± 1.24 ^**^
Nestin	54.01 ± 8.41	20.15 ± 2.64 ^*^	0.81 ± 2.62 ^**^
Differentiation of hADSCs in ND subjects			
CD34^(+)^	1.82 ± 1.72	0.08 ± 1.21	0.71 ± 1.05
CD45^(+)^	3.69 ± 1.88	0.90 ± 1.14	0.11 ± 1.92
CD73^(+)^	79.49 ± 3.42	4.06 ± 2.66 ^**^	2.90 ± 2.03 ^***^
CD90^(+)^	93.51 ± 3.01	0.73 ± 1.40 ^***^	0.98 ± 1.74 ^***^
CD105^(+)^	86.28 ± 6.76	13.53 ± 5.18 ^**^	5.04 ± 7.18 ^**^
Annexin V^(−)^/PI^(−)^/7AAD^(−)^ (viable cells)	97.12 ± 1.13	93.10 ± 2.44	98.33 ± 2.91
Annexin V^(+)^/PI^(−)^/7AAD^(−)^ (early apoptotic cells)	1.96 ± 0.76	4.71 ± 1.82	0.02 ± 2.16
Annexin V^(+)^/PI^(+)^/7AAD^(+)^ (late apoptotic, necrotic cells)	0.92 ± 1.33	2.19 ± 1.37	1.65 ± 0.24
NeuN	0.37 ± 1.09	45.20 ± 2.07 ^**^	50.31 ± 2.53 ^**, §^
TUJ1	1.94 ± 1.13	40.11 ± 4.17 ^**, §^	52.65 ± 6.24 ^**, §§^
O4	4.13 ± 5.10	38.14 ± 5.23^**^	18.04 ± 1.88 ^*, §^
GFAP	34.05 ± 3.67 ^§^	4.19 ± 2.12 ^**^	2.43 ± 4.16 ^**^
Nestin	73.56 ± 7.82 ^§^	16.20 ± 3.75 ^**^	1.05 ± 2.35 ^**^

Cells were labeled with fluorescence-coupled antibodies against CD34, CD45, CD73, CD90, CD105, Annexin V, 7AAD, propidium iodide solution (PI) and neural markers and analyzed using a MACSQuant flow analyzer as indicated in Methods. Results presented as mean ± SEM and presented as the percentage of cell surface marker per cell type (% of total cells) of all subjects each performed in duplicate in each group. Cells collected at day 0 before the induction of the differentiation (MSCs) and days 30 and 37 of differentiation (NSPs and NLCs, respectively)

hADSC human adipose-derived mesenchymal stem cell, MSC mesenchymal stem cell, ND neurodegenerative disease, NLC neural-like cell, NSP neurosphere-like structure

^*^P < 0.05, ^**^P < 0.01 and ^***^P < 0.005: NSPs/NLCs vs MSCs

^§^*P* < 0.05, ^§§^*P* < 0.01: ND vs healthy

To evaluate the neurogenic fate, the expression of neurogenic markers (TUJ1 and NeuN for neurons, O4 for oligodendrocytes, GFAP for astrocytes and nestin for undifferentiated MSCs) was evaluated by flow cytometry. First, the evaluation was realized on a mixed population without any purification. Double and triple labeling of the single-cell suspension of the same sample was realized each time to determine the degree of heterogeneity in the cell types present in the cultured samples. All of the analyzed data from the double (TUJ1-O4, TUJ1-GFAP, NeuN-O4, NeuN-GFAP, TUJ1-NeuN) or triple (NeuN-O4-GFAP, TUJ1-O4-GFAP, TUJ1-NeuN-O4, TUJ1, NeuN, GFAP) labeling confirmed the results presented in Table 1: we observed marked increases in the expression of NeuN, TUJ1 and O4 at day 30 and day 37, with a concomitant decrement in the expression levels of GFAP and nestin, indicating predominant neuronal and oligodendrocyte phenotypes but an inhibition of astrocyte differentiation. Significant lower potential of Neu-*Dif* was shown in ND subjects. These results were confirmed by the purification assays: we purified separately TUJ1^(+)^ cells from O4^(+)^ cells as well as from GFAP^(+)^ cells of MSC (day < 0) and NLC (day 37) populations (Table 2). The population of TUJ1^(1)^ was largely expressed in NLCs in comparison to O4^(+)^ and GFAP^(+)^, where TUJ1^(+)^ was increased markedly (~ 76%) with a concomitant moderate increase of O4^(+)^ (~ 21%) contrary to GFAP^(+)^ which decreased in NLCs (< 1%), particularly of the healthy group. Interestingly, in comparison to the healthy group, the ND group showed significant differences in terms of a lower percentage of cells expressing TUJ1^(+)^ (~ 31%) and O4^(+)^ (~ 10%), contrary to the higher percentage of GFAP^(+)^ cells (~ 8%). Importantly, the expression of ATP6AP2 in these purified cell populations showed significant variation between healthy and ND-derived cells only in the case of TUJ1^(+)^ population, the differentiated neuronal-like cells (18-fold vs 6-fold increases at day 37, healthy vs ND, respectively) (Table 2).

**Table 2:** Distribution of TUJ1^+^/O4^+^/GFAP^+^ purified cells before and after hADSC differentiation, and their relative mRNA ATP6AP2 expression

	Immunophenotyping^a^ (% of total cells)		ATP6AP2 mRNA^b^ (fold expression relative to MSCs)
	MSCs Day < 0	NLCs Day 37	NLCs Day 37
Healthy			
TUJ1^(+)^	2.87 ± 0.14	76.04 ± 0.22 ^***^	18.2 ± 0.49 ^***^
O4^(+)^	5.10 ± 0.38	21.12 ± 0.53 ^**^	4.27 ± 0.80 ^*^
GFAP^(+)^	20.92 ± 0.46	0.91 ± 0.65 ^***^	1.31 ± 0.42
ND			
TUJ1^(+)^	1.94 ± 0.90	31.44 ± 0.95 ^**, §§^	6.11 ± 0.83 ^*, §§^
O4^(+)^	1.08 ± 0.67	10.08 ± 0.48 ^*, §^	5.09 ± 0.78 ^*^
GFAP^(+)^	32.16 ± 0.71 ^§^	8.23 ± 0.51 ^*, §^	1.07 ± 0.95

hADSC human adipose-derived mesenchymal stem cell, MSC mesenchymal stem cell, ND neurodegenerative disease, NLC neural-like cell, NSP neurosphere-like structure

^a^2 × 10^7^ cells derived each from healthy or ND subjects were applied to purification at day < 0 and day 37 as indicated in Methods. Three positive fractions were purified separately: TUJ1^(+)^, O4^(+)^ and GFAP^(+)^ cells. Cells were labeled with anti-TUJ1-FITC or anti-O4-APC or anti-GFAP-PE and analyzed using a MACSQuant flow analyzer as indicated in Methods. Results presented as mean ± SEM of two independent experiments each performed in duplicate

^b^Expression of ATP6AP2 mRNA was determined by RT-qPCR on the purified fraction. Results expressed as fold variation relative to MSCs after normalization to GAPDH. Cells were collected at day 0 before the induction of the differentiation (MSCs) or at day 37 of the differentiation (NLCs)

^*^P < 0.05, ^**^P < 0.01 and ^***^P < 0.005: NLCs vs MSCs

^§^*P* < 0.05, ^§§^*P* < 0.01: ND vs healthy

MSCs and NLCs (at day < 0 and day 37, respectively) derived from healthy hADSCs were then stained with the neuronal marker TUJ1 and evaluated by confocal microscopy: we remarked an absence of TUJ1 in MSCs contrary to its high detection in differentiated NLCs (Fig. 2b). The heterogeneity of the differentiated population was confirmed by triple labeling of the single-cell suspension of the differentiated hADSCs at day 37 by staining TUJ1/O4/GFAP (Fig. 2c); it is important to note that the GFAP-labeled cells were nearly absent from this population.

### ATP6AP2 regulates p-ERK during neural differentiation

**Fig. 3:**
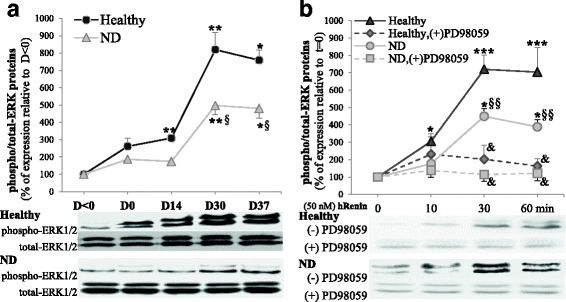
ATP6AP2 signaling is dependent on MAPK in hADSCs. hADSCs isolated from healthy and ND subjects and differentiated for 37 days. Cell lysates (80–150 μg of protein) separated by SDS-PAGE and immunoblotted with antibodies raised against p-ERK and total ERK. Proteins expression determined during differentiation from day < 0 to day 37 (**a**) or in NLCs at day 37 to evaluate the renin responsiveness (**b**). NLCs at day 37 treated after serum starvation overnight with human recombinant renin (hRenin, 50 nM) for indicated time course with or without 30 μM PD98059; to prevent any ERK phosphorylation potentially due to ANG II generation, Losartan (10 μM) and PD123319 (10 μM) ANG II blockers added during time course; quantitative variations in total and p-ERK amounts of p42 and p44 combined and shown. *^§^*P* < 0.05,**^§§&^*P* < 0.01,***P < 0.005: *D0/14/30/37 vs day < 0 or hRenin T10/30/60 vs *T* = 0, ^§^ND vs healthy, ^&^PD98059-treated vs untreated cells. D day, ERK extracellular signal-regulated kinase, ND neurodegenerative disease, *T* time

ERK had been confirmed as a key mediator of the ATP6AP2 signaling and can be activated by hRenin [5, 30]. Then, we checked the expression levels of total and phosphorylated ERK (p-ERK) proteins during neural differentiation. We observed (Fig. 3a) that total ERKs did not vary significantly between healthy and ND groups but p-ERKs increased. hRenin induced significant p-ERK particularly in healthy NLCs (Fig. 3b). These effects were abolished when cells were treated with PD98059 (10 μM), which is a selective inhibitor of MEK1/2 (the direct upstream kinase activator of ERK1/2) that inhibits ERK1/2 phosphorylation (Fig. 3b), indicating that ERK is required for the ATP6AP2 action.

### Involvement of ATP6AP2 in Wnt signaling responsiveness in NLCs

**Fig. 4:**
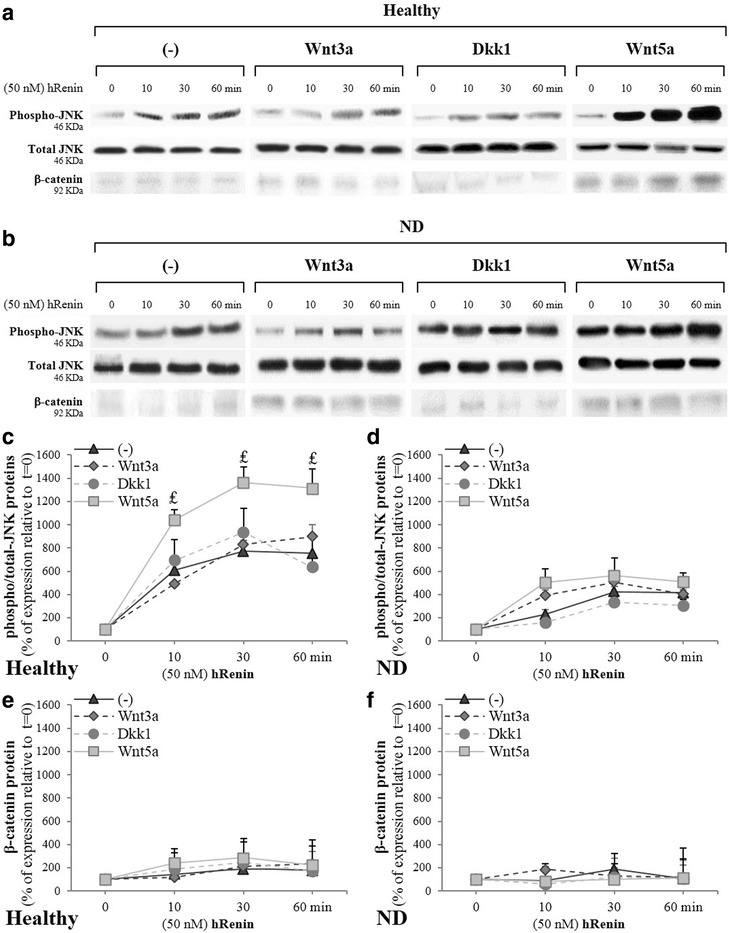
ATP6AP2 signaling is dependent on Wnt(s) signaling in hADSCs. hADSCs isolated from healthy and ND subjects and differentiated for 37 days. Cell lysates (80–150 μg of protein) separated by SDS-PAGE and immunoblotted with antibodies raised against p-JNK, total JNK1 and β-catenin. Role of renin and canonical/noncanonical Wnt signaling evaluated. NLCs at day 37 treated after serum starvation overnight with human recombinant renin (hRenin, 50 nM) as indicated in Fig. 3. NLCs at day 36 treated overnight with Wnt3a (50 ng/ml, 24 h) or Wnt-antagonist DKK1 (200 ng/ml, 24 h) and Wnt5a (100 ng/ml, 24 h), and at day 37 with hRenin (50 nM, 0–1 h). Representative blots (**a**, **b**) and quantitative variations (**c**–**f**) shown. ^₤^*P* < 0.01, Wnt5a-treated vs untreated cells. hRenin human recombinant renin, JNK Jun N-terminal kinase

Wnt signal pathways play key roles in neural differentiation [31]. To assess the Wnt signaling responsiveness, differentiated AD-NLCs were treated with canonical Wnt agonist (Wnt3a)/Wnt antagonist (Dkk-1) or noncanonical ligand (Wnt5a) for 24 h before adding hRenin as previously: p-JNK and translocation of β-catenin into the nucleus was evaluated. In fact, Wnt5A had been reported to activate Wnt/PCP signaling, which leads to the phosphorylation of JNK and its downstream target c-Jun [32, 33], whereas Wnt3A activates Wnt/β-catenin signaling by inducing its translocation into the nucleus [34]. Our results indicated in differentiated AD-NLCs a noncanonical Wnt signaling responsiveness involving ATP6AP2, not canonical signaling (Fig. 4).

First, hRenin induced efficiently p-JNK, but significant lower levels were seen in ND (Fig. 4a, b); total JNK did not vary significantly. This induction of phosphorylation has been enhanced strongly by Wnt5a; however, Wnt3a and Dkk1 failed to enhance the hRenin-induced JNK responsiveness (Fig. 4c, d). Second, hRenin failed to significantly induce β-catenin translocation into the nucleus (Fig. 4a, b); In fact, it was very difficult to detect the β-catenin protein in the nucleus or in the cytoplasm. The identification of the very modest levels in the nucleus allowed us to distinguish that there were no significant variations (Fig. 4e, f).

### Induction of ATP6Ap2 is accompanied with a transition from Wnt/β-catenin to Wnt/PCP signaling, and from CAV to FLOT

**Fig. 5:**
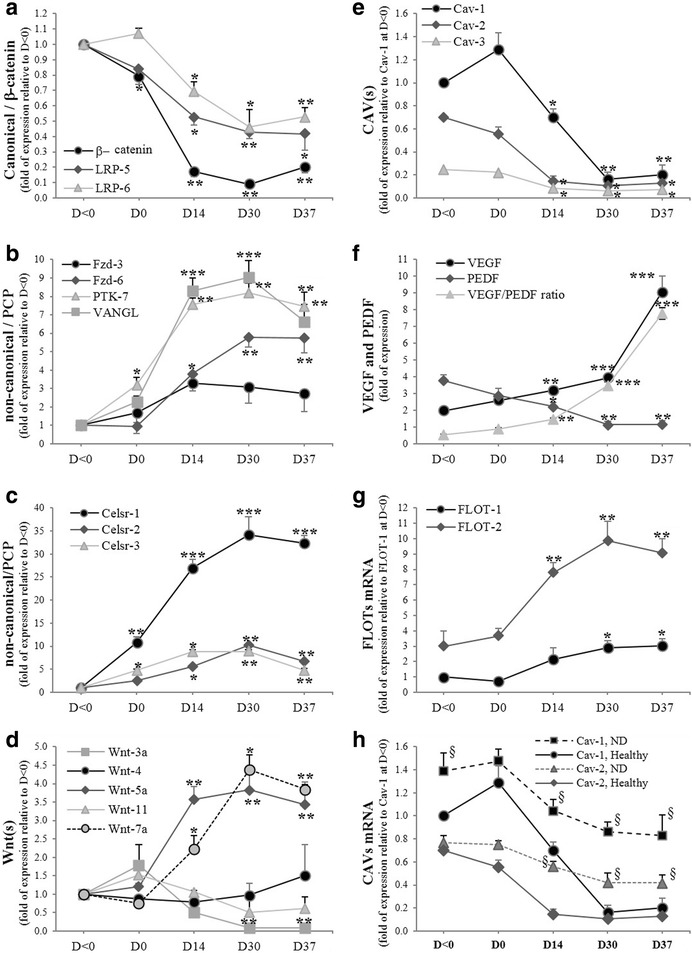
Transition of Wnt signaling responsiveness from Wnt/β-catenin to Wnt/planar cell polarity (PCP) during Neu-*Dif*. hADSCs cultured and differentiated as indicated in Methods; mRNA expression determined by RT-qPCR and normalized to GAPDH. Expression levels of canonical and noncanonical genes (β-catenin, LRP5 and LRP6 (**a**), Fzd-3, Fzd-6, PTK-7 and VANGL (**b**), Celsr(s) (**c**), Wnt(s) (**d**)), and caveolar/lipid raft key genes (CAVs (**e**), FLOTs (**f**)) and target gene of ATP6AP2 (VEGF/PEDF (**g**)) for healthy group. CAV-1/CAV-2 expression in ND group (**h**). *^§^*P* < 0.05,**P < 0.001,***P < 0.0001: *D0/14/30/37 vs day < 0, ^§^ND vs healthy. CAV caveolin, D day, FLOT flotillin, PEDF pigment epithelium-derived factor, VEGF vascular endothelial growth factor

The very low expression levels of β-catenin in differentiated NLCs led us to check its expression in undifferentiated cells. Therefore, the sequential activity of Wnt/β-catenin and Wnt/PCP signaling was examined during neural differentiation of ADSCs. Our results showed an attenuation of the Wnt/β-catenin pathway during neural differentiation whereas Wnt/PCP was unregulated. Upon differentiation, expression levels of canonical components such as β-catenin, LRP-5 and LRP-6 were gradually and significantly downregulated (Fig. 5a). Concurrently, the major noncanonical Wnt/PCP components such as Fzd-6, PTK-7 and VANGL (Fig. 5b) and the core PCP genes Celsr1/2/3 were continually and significantly upregulated (Fig. 5c). Simultaneously, Wnt-5a and Wnt-7a were markedly and significantly increased, contrary to Wnt-3a which was downregulated (Fig. 5d). No significant changes were noted in the mRNAs levels of Fzd-3, Wnt-4a and Wnt-11.

The attenuation of Wnt/β-catenin signaling could be related to a modulation in the expression of CAV, a major component of the plasma membrane microdomains where the Wnt signaling cascade can be initiated. In fact, it has been reported that CAV-1 plays a crucial role in inhibiting neuronal and oligodendroglia differentiation of neural stem cells in vitro through inhibition of β-catenin expression [35] and via a VEGF signaling pathway [14]. In addition, VEGF and ERK have been considered previously as downstream effectors of ATP6AP2 in endothelial cells, and treatment with hProrenin increases mRNA expression of VEGF and phosphorylation of ERK [11]. Thus, we determined the expression of CAV-1/2/3 as well as VEGF. We observed that CAV-1/2/3 were highly expressed in undifferentiated ADSCs and their mRNA expressions were dramatically attenuated during neural differentiation (Fig. 5e). CAV-1 was the most expressed isoform of CAV, whereas VEGF mRNA expression increased during neural differentiation contrary to its antagonist PEDF (Fig. 5f). These results are concomitant with those of β-catenin and the observed increases in p-ERK, and confirm the potential negative role of CAV-1 in inhibiting Neu-*Dif*.

However, the significant lack of CAV-1 in AD-NLCs suggests that other markers of lipid raft microdomains might be essential to the late phase of the neural differentiation. Among these components, FLOT belongs to a larger class of markers expressed in cells that lack CAV (e.g. neurons) [20], suggesting that it may represent a functional analogue of CAV-negative cells. As expected, the expression of FLOT-1 and FLOT-2 mRNAs increased during neural differentiation (Fig. 5g).

The profile of results obtained for the ND group was similar to that of healthy subjects but with moderate variations (data not shown), except CAVs which were not completely attenuated during neural differentiation (Fig. 5h).

### Subcellular distribution of ATP6Ap2 protein

The transition of expression from CAV to FLOT in AD-NLCs led us to check whether the subcellular distribution of ATP6AP2 could be affected. Cells were subjected to isolation of detergent-resistant microdomains (CLR-Ms).

**Fig. 6:**
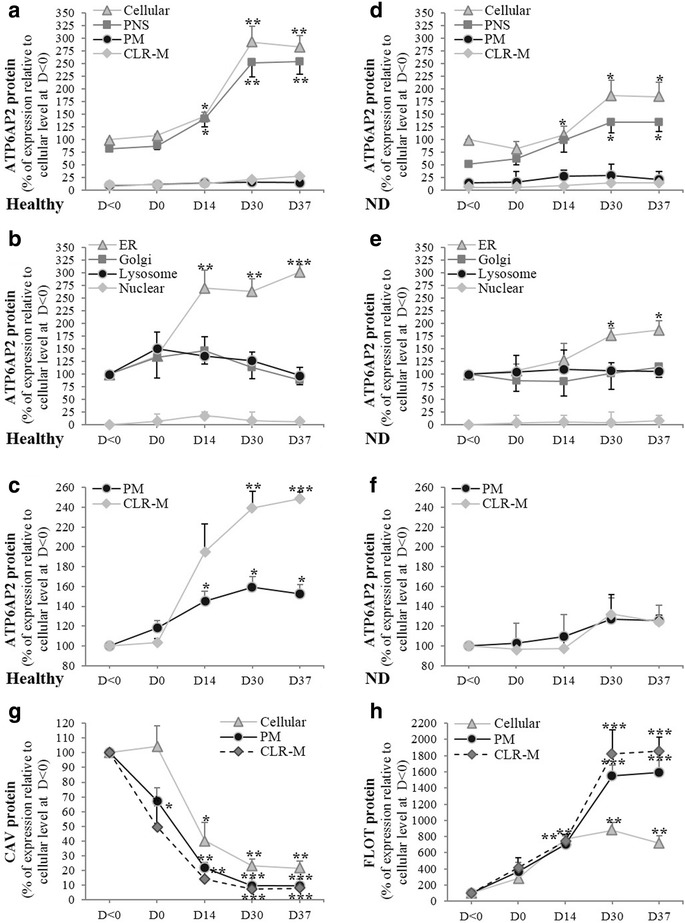
Subcellular distribution of ATP6AP2 proteins. Subcellular distribution of ATP6AP2 protein in cells derived from healthy (**a**–**c**) and ND (**d**–**f**) subjects examined during neurogenic differentiation of hADSCs during 37 days of differentiation. Lysate proteins (50–100 μg) separated by SDS-PAGE and immunoblotted with polyclonal antibodies directed against full length of ATP6AP2, N-20 recognizes CAV-1 and A-19 epitope mapping within internal region of FLOT-2. Subcellular distribution of ATP6AP2 determined in cellular (total lysates), plasma membrane (PM), caveolae/lipid raft membrane microdomain (CLR-M), postnuclear supernatant (PNS), endoplasmic reticulum (ER), Golgi, lysosome and nucleus. Expression of CAV (**g**) and FLOT (**h**) proteins during differentiation of healthy ADSCs. *P < 0.05,**P < 0.01;***P < 0.0005: D0/14/30/37 vs day < 0. CAV caveolin, D day, FLOT flotillin, ND neurodegenerative disease

The data demonstrated that ATP6AP2 is mainly localized intracellularly (Fig. 6a). Cell fractionation with various organelle markers showed colocalization of ATP6AP2 with the ER marker (TRAPα) but little colocalization was observed with the Golgi (TGN38, GM130), endosomal (EEA1) and lysosomal (LAMP1) markers and total absence with the nuclear (Nucleoporin, Lamin A) markers (Fig. 6b), indicating that ATP6AP2 is indeed highly concentrated in a mixed membrane compartment including ER and endosomes. Interestingly, parallel increases were seen also in the plasma membrane (Fig. 6c), particularly in CLR-Ms. In ND-derived cells, similar distribution was observed with lower levels of expression (Fig. 6d–f). The increases were accompanied with a significant drop of CAV at the cellular level, more importantly from PM and CLR-Ms (Fig. 6g), with strong increases of FLOT in CLR-Ms (Fig. 6h). Comparable subcellular distribution of CAV and FLOT was also observed in ND-derived cells (data not shown).

### GW4869 (GW) inhibits ATP6AP2, suspends its accumulation in CLR-Ms and reduces exosome release

CLR-Ms are plasmalemmal microdomains enriched in scaffolding proteins, but also in sphingolipids and cholesterol. We hypothesized that any disruption of these microdomains may affect ATP6AP2. Pharmacological treatments were used to achieve this goal targeting the mass of cholesterol or sphingolipids in CLR-Ms. First, we tried to deplete cholesterol by treating AD-MSCs with the most commonly used methyl-β*-*cyclodextrin (MBCD), a specific cholesterol-binding agent. MBCD (0–100 mM) was added for 48 h to adherent cells at day −2. Unfortunately, we did not succeed in maintaining the cells in adherence and viable along the whole process of differentiation. With doses superior or equal to 100 nM, the cytotoxicity effects and the apoptotic index were observed from the 4th day of differentiation depending on the utilized dose. Inferior to this dose, it was impossible to decrease significantly the cholesterol levels from CLR-Ms. Other cholesterol sequestering agents were used, nystatin and filipin: the disruption of CLR-Ms was optimal but the same problem as that of MBCD occurred. Cholesterol biosynthesis inhibitors (AY 9944, Triparanol) led to a marked reduction in cholesterol content, although here it was impossible to achieve the complete neural differentiation (success up to 9–11 days). However, when analyzing the expression of ATP6AP2 during these days, we observed intracellular increase in the ATP6AP2 protein levels (data not shown).

Second, the other main components of CLR-Ms are sphingolipids (sphingomyelin and glycosphingolipid)*.* Sphingomyelinases (SMase), ceramidases and glycosyl ceramide synthase (GCS) are key enzymes of sphingolipid metabolism that regulate the formation and degradation of ceramide. Inhibition of ceramidase (by D-e-MAPP, 10 μM, 48 h (day –2/0)) and GCS (by DL-1-PPMP, 20 μM, 48 h (day –2/0)) to increase the levels of ceramide had no cytotoxic effects and maintained cells in differentiation without significant loss (12–17%), causing sequential CLR-M depletion of CAV and accumulation of FLOT with moderate positive regulation of ATP6AP2. Complete blockage of ceramide synthesis by inhibiting the serine palmitoyltransferase (by ISP-1, 2 μM, 48 h (day –2/0)) had opposite effects but not potently (data not shown). Therefore, we aimed to block the activity of SMase: gluthatione GSH treatment (10 mM, 48 h (day –2/0)) by inhibiting N-SMase had elevated cytotoxic effects starting at day 5–7 of the differentiation (viability ranged from 23 to 45%); when ADSCs were treated with lower doses (order of micromolar and nanomolar), the viability of the cells started to be affected at day 11–12 (data not shown). Only the specific N-SMase inhibitor GW4869 (GW) treatment (20 μM, 48 h (day –2/0)) was efficient, without significant loss of cells (viability > 93%, apoptotic index < 3.8%), and achieving complete neural differentiation. GW induced significant accumulation of sphingomyelin in the plasma membrane, particularly in CLR-Ms with maximum effect at days 30 and 37. In the following section of this manuscript, we will present the data obtained in GW-treated ADSCs.

**Fig. 7:**
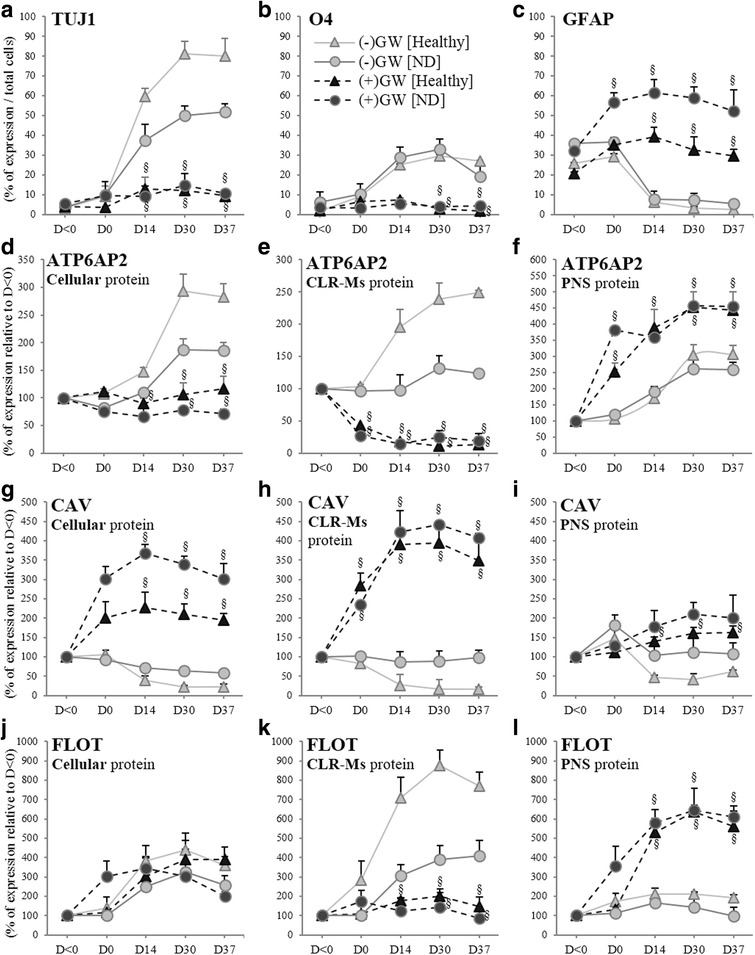
Disruption of CLR-M by GW4869 (GW) induces accumulation of ATP6AP2 protein in subcellular compartments. hADSCs derived from healthy and ND subjects differentiated with or without GW (20 μM, pretreated D-2/0). Expression of neural markers (TUJ1 (**a**), O4 (**b**) and GFAP (**c**)), and subcellular distribution (of ATP6AP2 (**d**–**f**)/CAV (**g**–**i**)/FLOT (**j**–**l**) proteins in cellular, CLR-Ms and postnuclear supernatant (PNS)). ^§^*P* < 0.01: GW^+^ vs GW^−^. CLR-M caveolae/lipid raft membrane microdomain, D day, FLOT flotillin, ND neurodegenerative disease

Our results showed that in GW-treated cells, neuronal TUJ1 and oligodendrocyte O4 markers were downregulated contrary to the astrocyte GFAP (Fig. 7a–c). Concurrently, considerable decreases in the levels of expression of ATP6AP2 protein (Fig. 7d–f) were shown particularly in CLR-Ms, but surprisingly accumulated in PNS, indicating a relocalization of ATP6AP2 in intracellular compartments. Thus, GW reversed successfully the expression of ATP6AP2. Disruption of CLR-Ms was reflected by a dynamic change in the abundance of CAV (Fig. 7g–i) and FLOT (Fig. 7j–l). GW induced increases in the levels of cellular CAV, strongly in CLR-Ms and slightly in PNS. No significant changes were observed in the cellular levels of FLOTs, but rather decreases in CLR-Ms indicating efficiently a reversible effect. However, FLOT accumulated significantly in intracellular compartments, indicating the likely reason why ATP6AP2 was increased in PNS after treatment with GW.

**Fig. 8:**
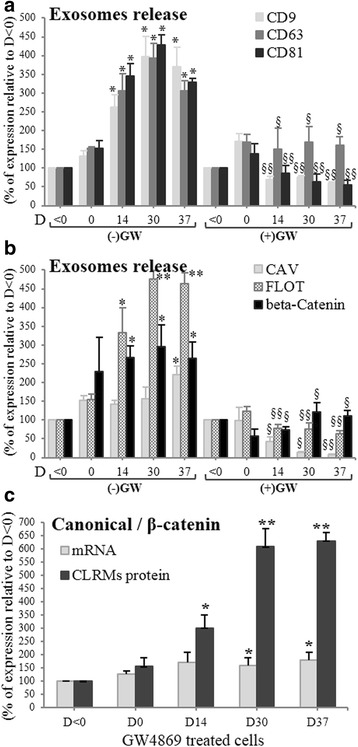
Disruption of CLR-M by GW4869 (GW) induces reduction of exosome release. hADSCs derived from healthy and ND subjects differentiated with or without GW (20 μM, pretreated D-2/0). Exosome fractions analyzed for the assessment of tetraspanins (CD9/63/81) (**a**) and FLOT/CAV/β-catenin (**b**). Expression of β-catenin mRNA and protein in CLRMs in GW-treated cells during Neu-*Dif* (**c**)*.* *P < 0.01, **P < 0.005: D0/14/30/37 vs day < 0; ^§^*P* < 0.01, ^§§^*P* < 0.005: GW^+^ vs GW^−^. CAV caveolin, CLRM caveolae/lipid raft membrane microdomain, D day, FLOT flotillin

Several reports have described release of exosomes from neural cells: differentiated [36] and developing neurons [37], oligodendrocytes [38] and astrocytes [39]. We hypothesized that in ATP6AP2-downregulated cells, the relocalization of ATP6AP2 concomitantly with FLOT, from the CLR-Ms into intracellular compartments, may be due to inhibition of exosome release. Several markers of the tetraspanin family including CD9, CD63 and CD81 are highly enriched in exosomal membranes; GW-pretreated cells significantly impaired release of exosomes during neural differentiation as observed by a significant reduction in the expression of CD9/CD63/CD81 (Fig. 8a), and the reduction of FLOT and CAV (Fig. 8b) recovered in the exosome fraction. These results collectively indicate that the disruption of CLR-Ms by GW affects exosome release and thus redistributes CLR-M components to intracellular sites. Finally, to know whether the Wnt signaling pathway can be affected, we investigated the effects of GW on the exosomal export of β-catenin. Consistent with the reduction in exosome release, a marked reduction in the release of β-catenin was also observed (Fig. 8b), indicating an accumulation of β-catenin in the ATP6AP2-downregulated cell. In fact, during Neu-*Dif*, the levels of β-catenin mRNA increased moderately; however, the levels of its protein in CLRMs increased significantly (Fig. 8c). No significant variations of CD9/63/81 were observed in GW-treated cells during Neu-*Dif* (data not shown). These results indicate that GW blocks exosome release by sequestration of β-catenin in the plasma membrane and not due to an effect at the transcriptional level.

### Gα proteins crosslink ATP6AP2 to CAV

**Fig. 9:**
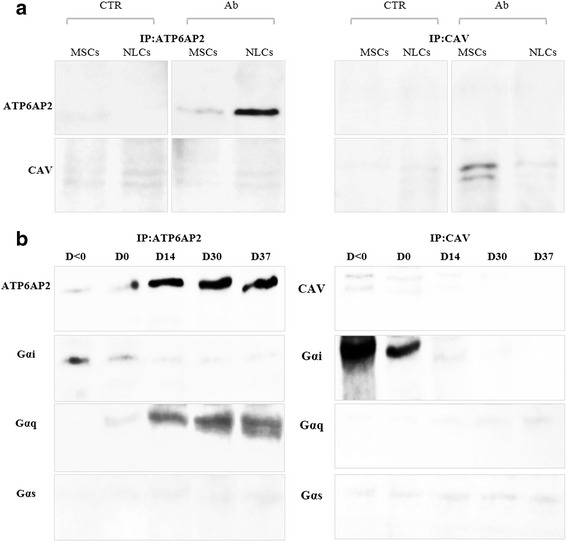
Interaction of CLR-Ms ATP6AP2/CAV and Gα proteins. hADSCs isolated from healthy subjects and differentiated for 37 days as indicated in Methods. Co-IP of ATP6AP2 and CAV in CLR-Ms: CLR-M fraction of MSCs and NLCs immunoprecipitated (IP) with anti-ATP6AP2 antibody and probed with anti-CAV1 antibody or vice versa (**a**). Co-IP of ATP6AP2 or CAV with G_α_ proteins (G_αs_, G_αi,_ G_αq_) in CLR-Ms (**b**). Input for whole experiments were positive. Representative blots of healthy group shown; similar results observed in ND group (data not shown). Ab antibody, CAV caveolin, CTR control, D day, MSC mesenchymal stem cell, NLC neuron-like cell

To further evaluate the interaction between ATP6AP2 and CAV, we performed co-IP experiments on CLR-Ms. Unexpectedly, no direct interactions were found between ATP6AP2 and CAV in CLR-Ms (Fig. 9a). These results led us to check by co-IP experiments whether any potential crosslink could occur between these two proteins and a third factor: the Gα proteins (Gαs, Gαi and Gαq). Importantly, Gαi and Gαq were significantly detected in CLR-Ms crosslinked with CAV and ATP6AP2 respectively, contrary to Gαs which was unchanged. During differentiation of ADSCs, we observed that IP:ATP6AP2 retained strongly Gαq in CLR-Ms of differentiated cells and moderately Gαi in CLR-Ms of undifferentiated cells, with no interaction with Gαs. However, IP:CAV retained strongly Gαi in CLR-Ms of undifferentiated cells and these interactions disappeared completely from CRL-Ms of differentiated cells, indicating a switch from Gαi to Gαq during differentiation (Fig. 9b). These concomitant increases of ATP6AP2 and Gαq were coupled to significant decreases of CAV and Gαi, indicating the eventual mechanism by which ATP6AP2 acts.

**Fig. 10:**
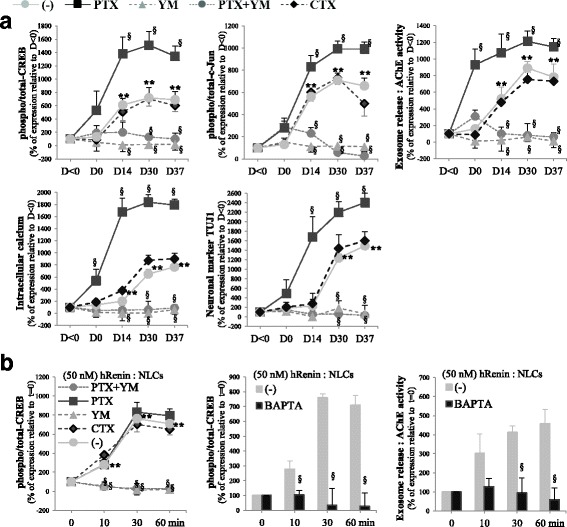
Activities of G_α_ protein-sensitive CREB/C-Jun, exosome releases and intracellular calcium during neural differentiation of hADSCs. hADSCs isolated from healthy subjects and differentiated for 37 days as indicated in Methods; cells treated for 48 h (day –2 to day 0) with G_αs_ activator cholera toxin (CTX, 1 μg/ml) or G_αq_ protein inhibitor YM254890 (YM, 1 μM) or G_αi_ inhibitor pertussis toxin (PTX, 100 ng/ml). Analyses performed along differentiation (**a**) or at NLC stage (day 37) (**b**). Nuclear extracts separated by SDS-PAGE and immunoblotted with antibodies directed against total and p-CREB and c-Jun. Secreted exosomes collected and quantitated by measuring AChE activity. Involvement of intracellular Ca^2+^ assessed by treating differentiated NLCs at day 37 with BAPTA-AM (25 μM, 24 h before adding hRenin) or by measuring [Ca^2+^]_i_ during Neu-*Dif* and using Fura2/AM labeling as described in Methods. Neu-*Dif* evaluated by TUJ1 protein expression. ATP6AP2 responsiveness to hRenin after CTX/PTX/YM treatments evaluated in NLCs at day 37. **P < 0.01: D0/14/30/37 vs day < 0. ^§^*P* < 0.01: treatments vs control. AChE acetylcholinesterase CREB cAMP response element-binding protein, D day, NLC neuron-like cell

To confirm the involvement of Gα proteins, we treated cells with the Gαs activator cholera toxin (CTX), the Gαq protein inhibitor YM254890 (YM) and the Gαi inhibitor pertussis toxin (PTX), and assessed the activation of the transcription factors CREB and c-Jun, major targets of ERK signaling in neuronal cells, and determined the exosome release. In control cells, the phosphorylation of CREB and c-Jun were induced during differentiation (Fig. 10). PTX increased significantly the nuclear p-CREB/c-Jun and the release of exosomes, whereas YM and YM + PTX abolished their activities during differentiation. The involvement of Gαq led us to assess whether the enhanced release of exosomes was due to an increase in intracellular Ca^2+^. Expectedly, [Ca^2+^]_i_ increased significantly during Neu-*Dif*; a strong increase was shown with PTX, whereas a blockade was obtained in the case of YM and YM + PTX (Fig. 10a). In addition, TUJ1 protein increased strongly in PTX-treated cells and decreased significantly in YM/YM + PTX-treated cells, indicating a crucial role of a switch from Gαi to Gαq for the induction of Neu-*Dif* (Fig. 10a). No significant changes were observed with CTX.

To assess the involvement of ATP6AP2, NLCs at day 37 were treated as described previously with hRenin; we observed a significant stimulation of p-CREB by hRenin and an inhibition of the phosphorylation by YM and YM + PTX, indicating the necessary role of Gαq for ATP6AP2 responsiveness (Fig. 10b). c-Jun and VEGF were modulated in a similar way (data not shown). Treatment of NLCs with the intracellular Ca^2+^chelator BAPTA-AM (which was added before hRenin) resulted in a significant inhibition of p-CREB as well as the release of exosomes, indicating a calcium-dependent mechanism (Fig. 10b).

### Suppression of ATP6AP2 by siATP6AP2 in hADSCs inhibits neurogenesis and enhances astrogliogenesis

**Fig. 11:**
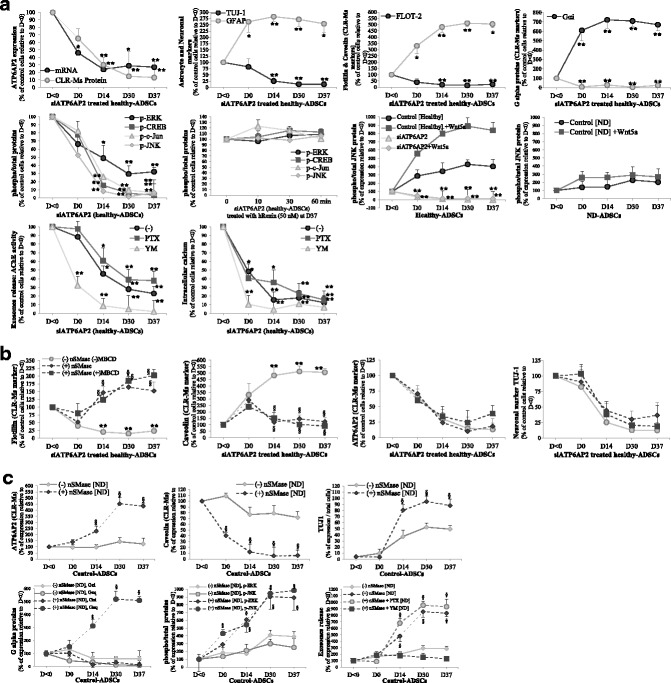
Inhibition of neurogenesis but enhancement of astrogliogenesis in siATP6AP2 cells and depletion of SM/CL failed to recover ATP6Ap2. **a**, **b** hADSCs derived from healthy subjects transfected with an ATP6AP2-targeting siRNA (siATP6AP2) for 48 h (day –2/0) and then differentiated with 1% FBS. **a** Evaluation of ATP6AP2 mRNA and CLR-M protein levels as well as several key factors during differentiation of ATP6AP2-knockdown cells from day < 0 to day 37: neuronal TUJ1 and astrocyte GFAP markers; CAV and FLOT proteins in CLR-Ms; G_αi_ and G_αq_ proteins in CLR-Ms; phospho-ERK1/2/CREB/c-Jun/JNK proteins. Obtained cells at day 37 treated with hRenin (50 nM) for indicated time course to evaluate renin responsiveness. siATP6AP2 cells also treated with G_αq_ protein inhibitor YM254890 (YM) or G_αi_ inhibitor pertussis toxin (PTX) as indicated in Fig. 10 to determine their impacts on exosome release and intracellular calcium concentration. **b** Impact of CLR-M disruption during siATP6AP2-hADSC differentiation derived from healthy subjects: at day 0, cells were treated with or without nSMase (1 mU/ml) and/or MBCD (10 nM) for 48 h*.* **c** Impact of nSMase on hADSCs derived from ND patients during Neu-*Dif*: ATPAP2 and caveolin proteins in CLR-Ms, expression of neuronal marker TUJ1, G_αi_ and G_αq_ proteins in CLR-Ms, phospho-ERK/JNK, and exosome release. *P < 0.05,**P < 0.01: siATP6AP2 vs control. ^§^*P* < 0.01: nSMase/MBCD vs control. ADSC adipose-derived mesenchymal stem cell, CAV caveolin, CLR-M caveolae/lipid raft plasma membrane microdomain, CREB cAMP response element-binding protein, D day, ERK extracellular signal-regulated kinase, FLOT flotillin, hRenin human recombinant renin, JNK Jun N-terminal kinase, MBCD methyl-β*-*cyclodextrin, ND neurodegenerative disease

Our data indicate that ATP6AP2 plays a key role synergically with CLR-Ms, but its direct role in Neu-*Dif* remains unclear. Thus we investigated whether ATP6AP2 is essential for neurogenesis of hADSCs: we transfected hADSCs derived from healthy subjects with an ATP6AP2-targeting siRNA (siATP6AP2) in comparison to a negative control siRNA, which has no targeting sequence. Several trials were done to get the high effectiveness of the transfection assay, and the best result was obtained when undifferentiated cells were treated for 48 h with siRNA (day –2/0) and then were differentiated as previously but with 1% FBS. We assessed the key factors in these ATP6AP2-knockdown cells (Fig. 11).

Figure 11a shows that mRNA and CLR-M protein levels of ATP6AP2 in cells transfected with siATP6AP2 were, indeed, significantly decreased; siATP6AP2 induced a significant decrease of TUJ-1-positive cells and increase of GFAP-positive cells. Taken together, these data suggest inhibition of neurogenesis and stimulation of astrogliogenesis. Concomitantly, siATP6AP2 abolished completely the FLOT expression in CLR-Ms contrary to CAV which accumulated abundantly. Furthermore, Gαq disappeared significantly from CLR-Ms whereas Gαi increased.

To further validate the function of ATP6AP2, we checked the expression of the phosphorylated key factors (ERK1/2, CREB, c-Jun and JNK), firstly during the differentiation of siATP6AP2 cells and secondly at day 37 in differentiated siATP6AP2-NLCs to evaluate the renin responsiveness: direct evidence of the impact of ATP6AP2 on ERK/CREB/c-Jun/JNK was observed; in fact, the phosphorylation of ERK1/2 as well as CREB/c-Jun/JNK decreased significantly during the differentiation of siATP6ATP2-cells and hRenin failed to induce their phosphorylation in siATP6AP2 NLCs, indicating that the factors are regulated by ATP6AP2. On the other hand, exosome release and the intracellular calcium concentration were inhibited in these cells during differentiation; in fact, when the cells were treated with the PTX and YM (Gαi and Gαq inhibitors, respectively), PTX did not reverse the inhibitory effects seen in these siATP6AP2 cells; however, YM had significant additional inhibitory effects on exosome release and intracellular calcium, indicating an essential role of Gαq in the mechanism of action of ATP6AP2: by silencing the ATP6AP2, Gαq is inhibited leading to inhibition of intracellular calcium and exosome release (Fig. 11a).

In Fig. 4, Wnt5a induced markedly p-JNK in response to hRenin in differentiated NLCs derived from healthy donors; here, we treated siATP6AP2 differentiated cells at day 37 with hRenin combined with Wnt5a and evaluated the levels of p-JNK: Wnt5a failed to enhance the hRenin-induced JNK responsiveness in these cells (data not shown). Furthermore, we treated healthy and ND-derived control and siATP6AP2 cells with Wnt5a 24 h before day 0 and maintained along the Neu-*Dif*: Wnt5a stimulated p-JNK strongly and significantly in healthy controls but failed to stimulate p-JNK in ND cells. Importantly, in siATP6AP2 cells, p-JNK was completely abolished and Wnt5a did not rescue its phosphorylation, indicating that ATP6AP2 is required for the Wnt5a-induced JNK responsiveness during Neu-*Dif* (Fig. 11a).

Next, to investigate whether a disruption of CLR-Ms may affect siATP6AP2 knockdown cells and induce any reversibility, and whether CLR-Ms without ATP6AP2 are responsible for the neurogenesis fate, we aimed to deplete membrane sphingomyelin and cholesterol after transfection of the cells with siATP6AP2*.* So, ADSCs were transfected from day –2 to day 0 with siATP6AP2 and then treated at day 0 with nSMase from *S. aureus* (1 mU/ml) and MBCD (10 nM) for 48 h (Fig. 11b). nSMase treatment of siATP6AP2 knockdown cells depleted CLR-Ms from CAV and induced accumulation of FLOT in these microdomains. However, no additional effects were shown when siATP6AP2 cells were treated with MBCD in addition to nSMase. Surprisingly, nSMase combined or not with BMCD did not recruit a significant amount of ATP6AP2 into CLR-Ms and did not promote neurogenesis in siATP6AP2-treated cells (no significant variations were observed for TUJ-1-positive cells), indicating a necessary role of ATP6AP2 for Neu-*Dif* and giving evidence that a reversibility in CAV levels in CLR-Ms may occur in cells not expressing ATP6AP2 without impacts on cell differentiation.

Based on these data, there is strong evidence that suppression of ATP6AP2 inhibits neurogenesis of hADSCs and a relocalization of ATP6AP2 into the CLR-Ms could remove the blockage of Neu-*Dif*. This may indicate that cells having significant high levels of ATP6AP2 in CLR-Ms with concomitant reduced levels of CAV should have high potential to promote neurogenesis. This has encouraged us to look at whether restoration of ATP6AP2 levels in the CLR-Ms of ADSCs derived from ND patients could re-stimulate their neurogenesis; therefore, we treated ND-hADSCs with nSMase as described previously and observed the following (Fig. 11c): ATP6AP2 accumulated in CLR-Ms where CAV decreased markedly with concomitant induction of the neuronal marker TUJ1; an important reversibility in the levels of Gαq occurred, contrary to Gαi which remained inhibited; a marked increase in the levels of phospho-ERK and phospho-JNK was seen; and, in addition, exosome release was induced significantly by nSMase treatment in these cells, where similar positive regulation was obtained in the case of treatment of ND-derived cells with nSMase + PTX (the inhibitor of Gαi), whereas YM (the inhibitor of Gαq) blocked completely the effects of nSMase. These results indicate that depletion of CLR-Ms from CAV, enrichment with ATP6AP2 and Gαq activity must be restored in ND-derived cells to induce Neu-*Dif.*

The dynamic changes in the plasma membrane microdomains led us to consider the importance of membrane fluidity in signal transduction and how it can affect mechanisms, thus we hypothesized that alterations in membrane fluidity may affect ATP6AP2-dependent neurogenesis and contribute to the development of neurological diseases. Cell membrane fluidity of ADSCs was assessed taking into consideration the relocalization of ATP6P2 from membrane to intracellular sites indicating the most important state where ATP6AP2 is no longer retained in the plasma membrane and caveolae. The cells were labeled with DPH probe, and fluorescence anisotropy was determined under conditions where DPH was primarily in the plasma membrane. The results indicate that the fluorescence anisotropy increased significantly when plasma membranes are deprived from ATP6AP2 (GW and siATP6AP2 treatments), indicating that the membrane is more rigid; however, an inverse situation was observed in ATP6AP2-enriched plasma membranes (nSMAse treatment), indicating that the membrane is more fluid which may activate the Gα signaling (Table 3).

**Table 3:** Membrane fluidity assessment

	Fluorescence anisotropy	Percentage of control	*P* value
MSCs			
Control (untreated cells)	0.184 ± 0.012	100	
GW	0.215 ± 0.019	116.8	< 0.05
nSMase	0.148 ± 0.008	80.4	< 0.01
siAT6AP2	0.219 ± 0.010	119.0	< 0.01
NLCs			
Control (untreated cells)	0.167 ± 0.009	100	
GW	0.208 ± 0.011	124.5	< 0.01
nSMase	0.150 ± 0.018	89.8	NS
siAT6AP2	0.203 ± 0.007	121.6	< 0.01

The cells derived from adipose tissue of healthy subjects were labeled with 1,6-diphenyl-1,3,5-hexatriene, and the fluorescence anisotropy of the probe was determined. The measurements were performed at 37 °C for 2 min immediately after addition of the fluorescent probe. Results presented as mean ± SEM

*P* < 0.05 and *P* < 0.001, treated cells compared with control cells

GW GW4869, MSC mesenchymal stem cell, NLC neural-like cell, NS not significant

## Discussion

ATP6AP2 ((P)RR) has been reported to be involved in neuronal development [40] and in neurogenesis and to be associated with neurological disorders [6, 41–43], but no previous study had clarified its role in the neurogenesis fate of hADSCs and its relation with CLR-Ms. In this study, we observed that (P)RR is expressed in ADSCs and increased during neural differentiation (contrary to s(P)RR), localized mainly intracellularly and active in CLR-Ms. Its activity is dependent on the CAV/FLOT balance. In differentiated cells, hRenin-induced (P)RR responsiveness of the MAPK pathway is accentuated by Wnt/PCP signaling. Concomitantly, a switch from canonical to noncanonical Wnt signaling occurs dependently on exosome release. In addition, Gα proteins crosslink ATP6AP2 to CAV, where a switch from Gαi to Gαq is necessary for the induction of the differentiation. Knockdown of ATP6AP2 inhibits neurogenesis and a relocalization of ATP6AP2 in CLR-Ms with concomitant reduced levels of CAV promotes neurogenesis.

Interestingly, cells derived from ND patients showed significant reduced levels of ATP6AP2 and downstream signaling. Recently, a meta-analysis of genome-wide association showed an inverse association between ATP6AP2 and multiple sclerosis, Alzheimer’s disease and Parkinson’s disease [44]. Previous reports have proved expression of ATP6AP2 in the brain and in neuronal cell differentiation [4, 5, 34], shown decreased levels in XPDS patients [6], shown that conditional depletion induced cognitive impairment and neurodegeneration [7] and reported gene mutation in patients with X-linked mental retardation and epilepsy [42].

Several studies have reported that ATP6AP2 is mainly localized in the ER, with minor concentration on the cell surface [3, 45]; our results supported these observations and showed an absence in lysosomes/nucleus and a dynamic localization in CLR-Ms. The accumulation of ATP6AP2 in CLR-Ms during Neu-*Dif* was concomitant with increased levels of FLOTs but decreased levels of CAVs, major CLR-M components. This indicates that cells having high amounts of CAV possess a nondifferentiated phenotype, but cells having high amounts of FLOT and ATP6AP2 possess the potential to differentiate into an NLC phenotype. FLOT is expressed during Neu-*Dif* [46] and is particularly high in cells that lack CAV (e.g., lymphocytes or neurons) [20]. Interestingly, CAV plays critical roles in regulating neuronal/oligodendral signaling pathways: downregulation of CAV-1 revealed more neuronal and oligodendroglia differentiation but not astrocytes [14, 35, 47], which is in accordance with our data observed with the siATP6AP2 cell model. Three CAV isoforms were expressed in AD-MSCs and nearly absent in AD-NLCs, and were shown previously to be expressed in brain endothelial and astroglial cell types [48]. CAVs may exert profound regulatory effects: indeed, CAV can negatively regulate the initiation of intracellular signaling by sequestering membrane signaling proteins (e.g., ATP6AP2, Wnt(s), V-ATPase, etc.) and preventing their interaction with the appropriate downstream signaling machinery. CAV-1 appears to positively regulate certain classes of signaling (β-catenin) [35] and attenuate others (ERK) [13], recruits β-catenin to caveolae in MDCK cells [49], and inhibits Neu-*Dif* in NPCs [14] via downregulation of VEGF, pERK, Akt and Stat3 signaling pathways, where VEGF signaling was a crucial target of CAV-1 [14]. Our results showed increased levels of VEGF mRNA and pERK during neural differentiation, and reduced levels in ND-derived cells. In addition, hRenin-induced pERK was completely abolished by the selective inhibitor of MEK1/2 kinase PD98059. In fact, previous studies underlie the combination role of ATP6AP2 and the VEGF in some neuropathogeneses [50, 51]. The role of VEGF is not limited to that of angiogenesis but also plays a crucial role in neurogenesis [10, 52]. Importantly, colocalization of ATP6AP2 and VEGF had been reported in hRECs, where hProrenin activates ERK signaling and increases the expression of VEGF mRNA, and the administration of ATP6AP2 blocker (NH2-RIFLKRMPSI-COOH) decreases the expression of VEGF [11]. Moreover, ATP6AP2 interacting with microRNA-152 regulates downstream VEGF expression in hRECs [12]. Interestingly, the mechanism by which ATP6AP2 acts is dependent on Gα proteins where an extinction of Gαi initiated the differentiation with a crucial role of Gαq for ATP6AP2-dependent neurogenesis: a mechanism linking intracellular calcium to exosome release and regulation of CREB and c-Jun, major targets of ERK signaling in neuronal cells. G-proteins relay signals from the plasma membrane to downstream effectors and the physiological functions of Gq and Gi have been largely reported previously in neural development and differentiation. Membrane fluidity seems to play an important role and initiate the ATP6AP2 signaling.

The finding that hRenin failed to induce translocation of β-catenin into the nucleus in AD-NLCs but induced the phosphorylation of JNK indicates clearly a noncanonical Wnt signaling responsiveness. These results were supported by the observation that Wnt-3a and Dkk1 failed to enhance hRenin response, contrary to Wnt5a. In fact, β-catenin/LRP-5/LRP-6 (the canonical Wnt components) were highly expressed in MSCs but significantly downregulated in NLCs, contrary to Fzd-3/PTK-7/VANGL/Celsr(s) (the major noncanonical Wnt/PCP components). In addition, Wnt-5a and Wnt-7a were markedly increased, contrary to Wnt-3a. Collectively, these results indicate a significant transition from Wnt/β-catenin to Wnt/PCP signaling during neural differentiation. Wnt signaling was involved in stem cell differentiation into neural lineages [31], and contradicts recently reported data in adult hippocampal neurogenesis [34] and in embryonic stem cells [53] which showed that Wnt/β catenin signaling blockade promotes neuronal induction and dopaminergic differentiation, and loss of LRP6 increases neuroectodermal differentiation. Furthermore, reduced distribution of LRP6 in the CLR-Ms (where CAV is associated) affects β-catenin signaling [54] and confirms that CAV binding to LRP6 is required upon Wnt stimulation [55]. ATP6AP2 forms a necessary link between LRP5/6 and Frizzled (Fz) [8].

Our results reveal a role of CLR-Ms in ATP6AP2 signaling, and emphasize the impact of CAV and FLOT. We previously reported that modulating sphingolipids at cellular levels [27, 56] and in microdomains [27] affects cholesterol, CAV and ERK signaling. Here, depleting sphingolipids from CLR-Ms showed increased expression of ATP6AP2 during neural differentiation, and sphingolipid enrichment reversed this situation. By inhibiting N-SMase with GW, neural differentiation of human ES cells was reversed [57]. In our study, GW inhibits neural differentiation: ATP6AP2 decreased and relocalized concomitantly with FLOT from the CLR-Ms into intracellular compartments, release of exosomes was impaired as observed by a significant reduction in the expression of markers of the tetraspanin family, the reduction of FLOT and CAV recovered in the exosome fraction and there was a reduction in exosome release of β-catenin. Previous reports showed that purified exosomes are enriched in ceramide and the release of exosomes was reduced after the inhibition of N-SMase [58]. A similar profile of results was reported previously where GW reduced exosome release in HEK 293 T cells [22], RAW264.7 macrophages [23] and proinflammatory cytokines [23]. siATP6AP2 knockdown ADSCs promoted astrogenesis but inhibited neuronal and oligodendrocyte differentiation, where ATP6AP2 disappeared from FLOT-deprived/CAV-enriched CLR-Ms: similar to GW-treated cells. These two cell models give evidence of the impact of a mutual association between ATP6AP2 and CLR-Ms on neural differentiation; in fact, disruption of CLR-Ms by GW accumulated sphingolipids/CAV in CLR-Ms but deprived them of ATP6AP2; and ATP6AP2 knockdown cells deprived CLR-Ms of ATP6AP2 but accumulated CAV. However, only the data obtained from nSMase-treated siATP6AP2 cells give strong evidence of the main role of ATP6AP2 in Neu-*Dif* to be maintained in CLR-Ms for neuronal induction. In fact, when siATP6AP2 cells are treated with nSMase, recovery of FLOT in CLR-Ms and depletion of CAV from CLR-Ms were observed without induction of the Neu-*Dif*, indicating that it is essential to relocate ATP6AP2 in CLR-Ms for neurogenesis.

In conclusion, this study shows that ATP6AP2 plays an important role in neural differentiation of ADSCs, and its activity involves the Wnt signaling pathways and Gα proteins in a way dependent on the dynamics of the membrane microdomains affecting the release of exosomes, thus giving evidence that its intracellular accumulation may be determinant for the induction of neuronal and oligodendrocyte differentiation. Our results provide an acceptable promise to use hADSCs as a proper autologous adult stem cell population for cell replacement therapy of neurological disorders, but highlight the importance in the selection of cells that must have higher amounts of ATP6AP2 particularly in CLR-Ms for better response, thus emphasizing the crucial role of CLR-Ms. For this purpose, further studies are required to test in vivo the hypothesis that in neurodegenerative disorders the autologous transplantation of undifferentiated stem cells must be considered carefully or instead will be needed prior to induction of neural differentiation. The role of ATP6AP2-over/underexpressed ADSCs should also be clarified during differentiation into other lineages of cells (hepatocytes, nephron progenitor cells, cardiomyocytes, etc.) in a perspective of cell therapy; for example, cardiovascular diseases where hypertension and hypercholesterolemia were correlated with the RAS components [59, 25], which in turn were associated with ATP6AP2.

## Conclusion

**Fig. 12:**
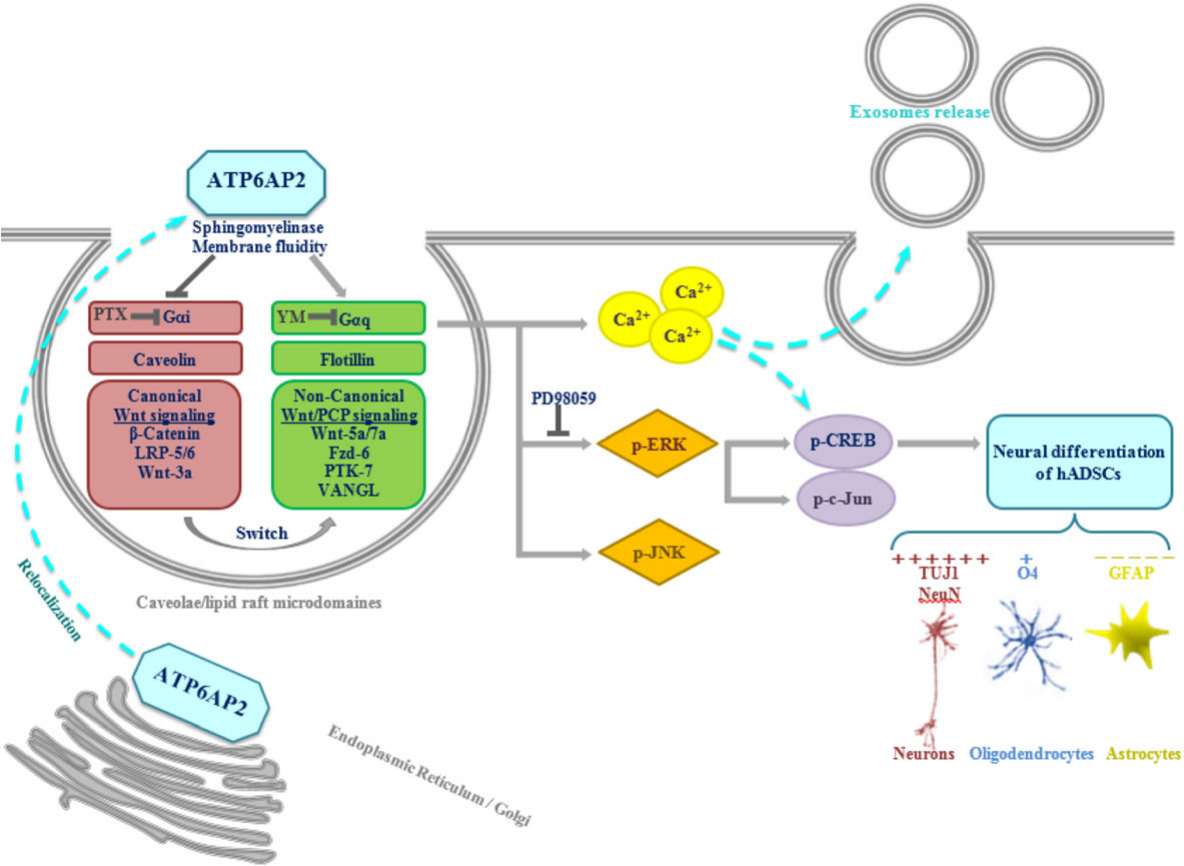
Model for regulation of ATP6AP2-induced neural differentiation by Wnt5a/Gαq/ERK/JNK/c-Jun/CREB and calcium-dependent exosome release signaling pathway

The novel observation of this report is that ATP6AP2 is associated with CLR-Ms and regulates the composition of the membrane microdomains, affecting membrane fluidity and mediating different Wnt signaling pathways by a mechanism which is dependent on a switch from Gαi to Gαq concomitantly with caveolin to flotillin, canonical to PCP (attenuation of β-catenin/LRP-5/LRP-6/Wnt-3a, increase of Fzd-6/PTK-7/VANGL/Celsr1–3/Wnt-5a/Wnt-7a, unchanged Fzd-3/Wnt-4a/Wnt-11) and intracellular Ca^2+^-dependent release of exosomes (Fig. 12). hRenin stimulates ATP6AP2 to induce p-ERK/JNK/VEGF/CREB/C-Jun and exosome release. ADSCs are competent to differentiate into neurons when CLR-Ms are enriched in ATP6AP2 and deprived of caveolin. Knockdown of ATP6AP2 which deprives CLR-Ms of ATP6AP2 inhibits neurogenesis but induces astrogenesis, and caveolin depletion did not recover the inhibition of Neu-*Dif*. Physiological relevance is found in patients with neurological disorders.

## Additional files

Additional file 1: **Table SI1.** Markers of nuclear and microsomal proteins in nuclear, microsomal and CLR-M fractions. (DOCX 75 kb)

Additional file 2: **Table SI2.** Primer sequences used for quantitative RT-PCR. (DOCX 78 kb)

## Abbreviations

(P)RR: Prorenin receptor
AChE: Acetylcholinesterase
AD: Alzheimer’s disease
ADSC: Adipose-derived mesenchymal stem cell
ALS: Amyotrophic lateral sclerosis
CAV: Caveolin
CD: Cluster of differentiation
CLR-M: Caveolae/lipid raft plasma membrane microdomain
CREB: cAMP response element-binding protein
DPH: 1,6-Diphenyl-1,3,5-hexatriene
ER: Endoplasmic reticulum
ERK: Extracellular signal-regulated kinase
FLOT: Flotillin
GAPDH: Glyceraldehyde 3-phosphate dehydrogenase
IP: Immunoprecipitate
JNK: Jun N-terminal kinase
MAPK: Mitogen-activated protein kinase
MLR: Microdomain lipid raft
MS: Multiple sclerosis
MSC: Mesenchymal stem cell
NB: Neurobasal
N-CLR-M: Noncaveolae/nonlipid raft plasma membrane microdomains
ND: Neurodegenerative disease
Neu-*Dif*: Neuronal differentiation
NLC: Neuron-like cell
NSC: Neural stem cell
NSP: Neurosphere-like structures
PBMC: Peripheral blood mononuclear cell
PD: Parkinson disease
PEDF: Pigment epithelium-derived factor
PM: Plasma membrane
PSA: Penicilin–stryptomycin–amphotericim
RAS: Renin–angiotensin system
SVF: Stromal vascular fraction
TGN: Trans-Golgi network
VEGF: Vascular endothelial growth factor
Wnt: Wingless-type MMTV integration site family member
