# Supplementary material for: Essential role of ATP6AP2 enrichment in caveolae/lipid raft microdomains for the induction of neuronal differentiation of stem cells
Source: Stem Cell Res Ther. 2018 May 11;9:132. doi: 10.1186/s13287-018-0862-9 (PMC5948768; doi:10.1186/s13287-018-0862-9)
Supplement: Supplementary file 1 — Table SI1. Markers of nuclear and microsomal proteins in nuclear, microsomal and CLR-M fractions. (DOCX 75 kb) [file 13287_2018_862_MOESM1_ESM.docx]

**Additional file 1**

**Table SI1.** Markers of nuclear and microsomal proteins in nuclear, microsomal and CLR-M fractions.

|  | Microsomal | Nuclear | N-CLR-M | CLR-M |
| --- | --- | --- | --- | --- |
| TGN38 | 100 % | 1.74±1.00 % | 2.63±0.92 % | 0.89±1.05 % |
| Nucleoporin p62 | 5.62±2.07 % | 100 % | 1.10±1.27 % | 0.77±1.12 % |

Equal amounts of protein (50 μg) were subjected to SDS/PAGE, immunoblotting and densitometric scanning (Gel Doc 2000 imaging system, Bio-Rad). The results are expressed as the amount of the marker protein relative to the indicated subcellular fraction (set to 100%). The experiments shown are representative of 3 independent experiments. *Abbreviations: non-caveolae /non-lipid raft plasma membrane (N-CLR-M), caveolae/lipid raft plasma membrane (CLR-M)*
