# Supplementary material for: Essential role of ATP6AP2 enrichment in caveolae/lipid raft microdomains for the induction of neuronal differentiation of stem cells
Source: Stem Cell Res Ther. 2018 May 11;9:132. doi: 10.1186/s13287-018-0862-9 (PMC5948768; doi:10.1186/s13287-018-0862-9)
Supplement: Supplementary file 2 — Table SI2. Primer sequences used for quantitative RT-PCR. (DOCX 78 kb) [file 13287_2018_862_MOESM2_ESM.docx]

**Additional file 2**

**Table SI2.** Primer sequences used for quantitative RT-PCR

| **Gene** | **Forward Primer 5’- 3’** | | **Reverse Primer 5’- 3’** |
| --- | --- | --- | --- |
| ATP6AP2 | | GAGAGGGCCTGAAGAGTGTG  GACACCTCCCTCATTAGG | CAGGCTGAGCCCAACTACTC  GCCAAGGCGATCATTATC |
| CAV-1 | | cacagttttcacggcatttg | gctggtgcactgaatctcaa |
| CAV-2 | | AGCAGGTGCTTGGCTTTTTA | TAACGGCAATGCTGAGACTG |
| CAV-3 | | ACAACACCCAGAGGAACCAG | AACCTGGCACTCATTTACGG |
| FLOT-1 | | GTGCAAAGGCATCTGACTGA | TTTGCAGGATTCAATGTCCA |
| FLOT-2 | | CTCTTCATGGGCATGTTCCT | CACTCTGGCCAGGAAGAAAG |
| Celsr-1 | | AGTGTGGGCCCAGTCACTAC | CACTGGCCGTTGGTCTTATT |
| Celsr-2 | | GTGACTCAAACCCGTGTCCT | CTCACAGTATGGCCCAAGGT |
| Celsr-3 | | GAACGAGAAGCACGACTTCC | GTCCGGGGCTTGTTGTAGTA |
| Fzd-3 | | ATTTCCGGCCTTTTCTTTGT | TCCATATCTTCAGGCCAAGG |
| Fzd-6 | | GAGCCATCAAGCAGTCACAA | GGGGCAGTAGGAAAAACACA |
| PTK-7 | | GTGGATCTGGGAGACCTCAA | TCACTTGTCTCTGGGCACTG |
| VANGL | | GCAAGACCAGCTTCCTTGAC | AGGAACCACAAAGCCAACAC |
| Wnt-3a | | CCACACCGTCAGGTACTCCT | TGTAGCTGGATGGAGTGCAG |
| Wnt-4 | | ACAGCTGGAAGGCTGACAGT | TGCATGTCCTTCTCACAAGC |
| Wnt-5a | | ATACTGGCTGACCACCTTGG | GACAAAGGCCTCAGAAGCAC |
| Wnt-7a | | CCCTGAAGCTTACTGCTTGG | GCTACGATGTATGGGGCACT |
| Wnt-11 | | CACCCCCAGATAGTTGTGCT | GAGGAGGAAAGCGACACAAG |
| β-catenin | | GAAACGGCTTTCAGTTGAGC | CTGGCCATATCCACCAGAGT |
| LRP-5 | | CCCAAACTGTCTGTCCTGGT | CCCAGCTGTGCATCACTAGA |
| LRP-6 | | TTCCCCTGGAGATTCTGATG | GGATGGAAAACCCCAGATTT |
| VEGF | | GCTGTCTTGGGTGCATTGG | GCAGCCTGGGACCACTTG |
| PEDF | | TTCAAAGTCCCCGTGAACAAG | GGATCGCACCCGGTACAG |
| GAPDH | | CATGAGAAGTATGACAACAGCCT | AGTCCTTCCACGATACCAAAGT |
